# Selection-aware metrics provide complementary decision-level information in plant genomic prediction

**DOI:** 10.3389/fpls.2026.1951182

**Published:** 2026-10-01

**Authors:** Xueyang Wang, Jun Yan

**Affiliations:** Frontiers Science Center for Molecular Design Breeding, State Key Laboratory of Maize Bio-Breeding, National Maize Improvement Center, College of Agronomy and Biotechnology, China Agricultural University, Beijing, China

**Keywords:** candidate selection, genomic prediction, multi-environment prediction, observed top-k recovery, Pearson correlation, standardized observed selection differential

## Abstract

Breeding programs increasingly need prediction systems that support reliable candidate selection across heterogeneous environments, which makes cross-environment genomic prediction an important component of multienvironment crop improvement. However, model evaluation still relies heavily on Pearson’s correlation coefficient (*r*), a global summary that may not fully capture which top-ranked candidates would be advanced. Using maize and switchgrass datasets as focused case studies, we compared global predictive ability with phenotype-referenced candidate diagnostics across within-environment and directed cross-environment evaluations of rrBLUP/ridge, Light Gradient Boosting Machine (LightGBM), a multilayer perceptron, and mixed-effect deep neural network (MeNet). Rather than proposing a new selection metric, we used a conditional comparison framework that distinguishes nominal metric reversal from strict decision-level discordance among models with similar Pearson’s *r*. The *r*-best and candidate-metric-best models disagreed in 25%–55% of evaluation contexts, but large differences in candidate metrics among near-*r* models (|Δ*r*| ≤ 0.02) were uncommon. Nevertheless, models with nearly identical *r* did not always prioritize the same candidates: LightGBM and rrBLUP/ridge shared, on average, 72.9% of the top 5% candidates in maize and 68.8% in switchgrass. These results indicate that Pearson’s *r* remains informative for broad model comparison but does not fully describe decision-level behavior. Phenotype-referenced candidate diagnostics are therefore best used as targeted complements when models have similar predictive ability, selection intensity is high, or candidate identity is consequential across environments.

## 1 Introduction

Genomic prediction has become a central tool in plant breeding because it enables early prediction of phenotypic performance or breeding value from genome-wide marker information. Since the original development of genomic selection (Meuwissen et al., 2001), ridge-type shrinkage models and mixed-model approaches have remained important baselines, while tree-based machine learning models and neural networks have expanded the range of prediction approaches available for high-dimensional marker data (Endelman, 2011; de los Campos et al., 2013; Ke et al., 2017; Yan and Wang, 2023; Crossa et al., 2025).

Breeding programs that evaluate material across multiple environments require prediction systems that support reliable selection under environmental heterogeneity. As genotype-by-environment interactions can alter both trait expression and candidate rankings, multienvironment genomic prediction is not only a problem of predictive ability but also of decision consistency: competing models may agree broadly while prioritizing different materials when decisions are transferred between environments.

The most common empirical summary of genomic prediction performance remains the Pearson’s correlation coefficient between predicted and observed phenotypes. This quantity is often called predictive ability and is useful because it is simple, scale-free, and comparable across datasets. However, it should not be automatically equated with true prediction accuracy in the quantitative-genetic sense, which would require the correlation between predicted and true genetic values or additional assumptions about phenotype reliability and heritability. Likewise, predictive ability is only one dimension of model evaluation; bias, dispersion, and calibration are established complementary concerns (Legarra and Reverter, 2018; Gowane et al., 2019).

A practical breeding decision is often not based on average agreement across the full population. Many stages of selection address a narrower question: which individuals should be advanced? Previous genomic prediction studies have therefore evaluated selected-set agreement or coincidence, ranking quality such as normalized discounted cumulative gain (NDCG), top-fraction recovery, gain-oriented summaries, and sensitivity/specificity-type criteria (Ornella et al., 2014; Blondel et al., 2015; dos Santos et al., 2020; Chen et al., 2024; Heinrich et al., 2025; Montesinos-Lopez et al., 2025). Collectively, this literature establishes that selection-oriented summaries can complement global predictive ability; the component metrics used here are not introduced as new metrics.

What remains less well characterized is a narrower model comparison question: when competing models are already nearly tied in global predictive ability, how often do they produce decision-level differences large enough to matter for candidate prioritization, and are the same candidates retained under directed cross-environment transfer? A strong claim that Pearson’s *r* systematically fails is not supported by our final data. Our conditional hypothesis is instead that global and candidate-level metrics tend to agree when model differences are large, but can diverge when models are closely matched, the retained fraction is small, or prediction is transferred across environments.

Accordingly, the contribution of this study is not the introduction of a new selection metric, but an empirical framework for determining when established phenotype-referenced candidate diagnostics provide information beyond global predictive ability. We make three specific contributions. First, we jointly evaluate Pearson’s *r* and error summaries with observed top-k recovery, standardized observed selection differential (OSD@k), and predicted candidate-set overlap within the same evaluation contexts. Second, we distinguish nominal model choice reversal from strict discordance among near-*r* model pairs and examine the upper-tail candidate turnover that gives rise to such cases. Third, we extend this conditional comparison to directed cross-environment prediction, where global transfer performance can be evaluated alongside the stability of candidate prioritization. The model set deliberately spans linear shrinkage, tree-based nonlinear learning, a generic neural network, and an environment-aware neural model. All candidate diagnostics are interpreted retrospectively against observed or adjusted phenotypes rather than as direct estimates of realized genetic gain. The overall evaluation framework is summarized in **Figure 1**.

**Figure 1:**
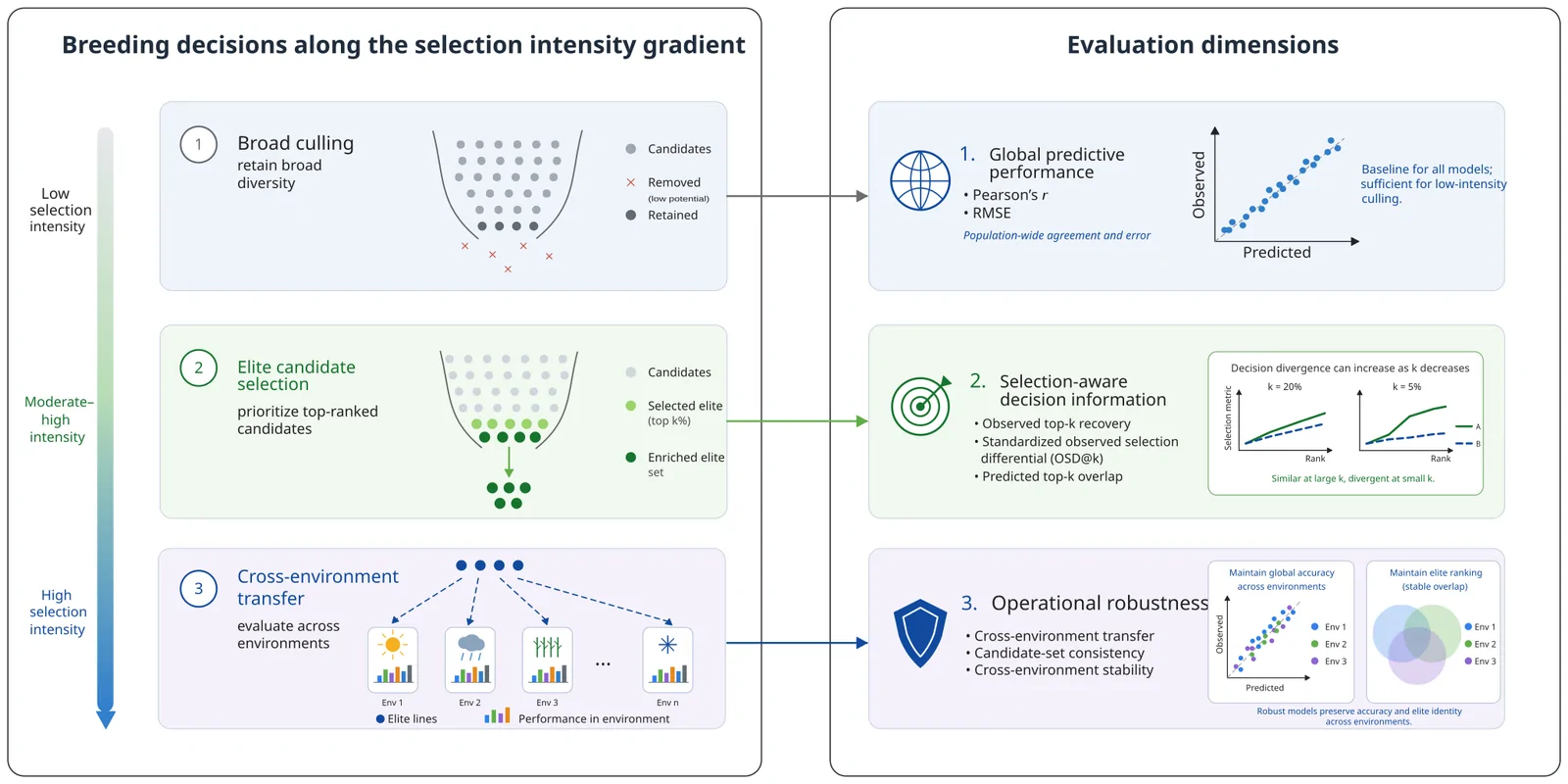
Conceptual framework used in this study to integrate established global predictive performance summaries with phenotype-referenced candidate diagnostics and directed cross-environment decision consistency. Pearson’s *r* and RMSE remain the primary global summaries, while observed top-k recovery, OSD@k, predicted top-k overlap, and cross-environment consistency provide targeted complementary diagnostics.

## 2 Materials and methods

### 2.1 Datasets and trait definitions

The final analysis used two within-environment prediction tasks and one maize cross-environment task (**Table 1**). We describe the study as a focused empirical evaluation in maize and switchgrass rather than as a broad multicrop benchmark. Additional analysis summaries and quality-control statistics for the processed inputs are provided in a single **Supplementary Information File**.

**Table 1:** Dataset and task summary.

Task	Crop/material	Trait used	Samples	Markers	QC summary	Evaluation	Source
Within-environment maize EW	Maize F1 hybrids from the NCII6210 resource	EW; processed from across-location BLUP table	5,831	5,000	Missing = 0.0; min MAF = 0.0539; median MAF = 0.230	5 repeats × 5-fold nested CV; rrBLUP/ridge, LightGBM, MLP	CUBIC-related NCII hybrid resource (Xiao et al., 2021; ZEAMAP/MaizeGo)
Within-environment switchgrass HT	*Panicum virgatum* Northern Switchgrass Association Panel	HT; extracted from ST/HT/AN phenotype table	514	10,000	Missing = 0.0; min MAF = 0.0545; median MAF = 0.455	5 repeats × 5-fold nested CV; rrBLUP/ridge, LightGBM, MLP	G3 genomic prediction benchmark (Azodi et al., 2019; Dryad); phenotypes from Lipka et al. (2014), genotypes from Evans et al. (2018)
Cross-environment maize EW	Maize F1 hybrids across BJ, HN, HeB, JL, and LN	EW; standardized within environment for directed cross-environment fitting/evaluation	Test sets: 5,749–5,829 hybrids per environment	5,000	Same processed maize marker set; min MAF = 0.0539	20 directed train–test environment pairs; rrBLUP, LightGBM, MLP, MeNet	Same NCII6210 hybrid resource; location-specific table Ncii_2015_5locs_hybrids.txt

EW, ear weight; HT, height; OSD, standardized observed selection differential. Marker statistics are from the final processed input matrices used for the analyses.

The maize task was derived from the CUBIC-related North Carolina II (NCII) hybrid resource reported by Xiao et al. (2021). That resource was generated by crossing maternal lines from the CUBIC population with 30 paternal testers. The original study measured days to tasseling (DTT), plant height (PH), and ear weight (EW) in F1 hybrids across five locations, and defined EW as the average weight of five fully formed ears in the middle of each plot row. For within-environment prediction, we used the processed EW phenotype from the across-location best linear unbiased prediction (BLUP) table (Ncii_2015_blup_hybrids.txt). For cross-environment prediction, we used the five-location table (Ncii_2015_5locs_hybrids.txt), which contains the columns loc, Lineid, DTT, PH, and EW for Beijing, Henan, Hebei, Jilin, and Liaoning. The processed maize within-environment dataset contained 5,831 hybrids and 5,000 genome-balanced SNP markers after marker selection and quality control. The marker matrix contained no missing values, a minimum MAF of 0.0539, and a median MAF of 0.2296 (**Supplementary Table S6**).

The switchgrass task was derived from the public G3 genomic prediction benchmark compiled by Azodi et al. (2019), with switchgrass phenotypes from Lipka et al. (2014), and genotype data came from Evans et al. (2018), based on the Northern Switchgrass Association Panel. The raw phenotype table used in this study contained ST, height (HT), and AN, corresponding to standability, height, and anthesis date. We analyzed HT as the target trait. The processed switchgrass dataset contained 514 individuals and 10,000 single nucleotide polymorphism (SNP) markers after quality control and marker selection. The processed marker matrix contained no missing values, a minimum MAF of 0.0545, and a median minor allele frequency (MAF) of 0.4553 (**Supplementary Table S6**). The processed HT phenotype was analyzed using the standardized scale present in the processed phenotype file.

Phenotype reliability information was taken from the corresponding source studies where it could be matched to the analyzed trait. For switchgrass, Lipka et al. (2014) reported a clone-mean repeatability of 0.75 (standard error [SE] = 0.03) for plant height across three summer environments. As the benchmark used here was subsequently reformatted and normalized by Azodi et al. (2019), and because the final processed sample intersection differs from the line count used for the original repeatability estimate, we treat this value as trait-level context from the source study rather than as an observation-specific reliability correction. For the maize NCII6210 hybrid EW task, the source study used multienvironment BLUP processing to reduce environmental variation, but we did not identify a directly matching numerical heritability or reliability estimate for the processed F1 hybrid EW response. We therefore do not apply a heritability-based correction for predictive ability for either task.

### 2.2 Genotype and phenotype preprocessing

For within-environment nested cross-validation, genotype matrices were matched to phenotype records by sample identifier, and marker columns were converted to numeric values. The final processed matrices used here had no remaining missing marker values. Within each training fold, phenotypes were standardized using the training-fold mean and population standard deviation, and the parameters for marker standardization for Ridge, LightGBM, and MLP were learned from the corresponding training data. Test-fold predictions were back-transformed using the training-fold phenotype mean and standard deviation before calculating Pearson’s *r*, RMSE, observed top-k recovery, OSD@k, and candidate-set overlap. Thus, within-environment model comparisons were made on the same original processed phenotype scale without using test phenotypes to fit the training transformation.

For directed maize cross-environment analyses, the source-environment phenotype was standardized using the source-environment mean and population standard deviation before model fitting, while the observed target-environment EW was independently standardized within the target environment for evaluation. Ridge and MLP used marker scaling fitted in the source environment and applied to target samples; the archived LightGBM cross-environment implementation used numeric marker inputs directly. Model predictions were retained on the source-standardized response scale and were not recalibrated using target-environment phenotypes. For MeNet input preparation, genotype states were recoded from 0/1/2 to − 1/0/1 and environment-standardized phenotype files were used for the final train, validation, and test inputs.

### 2.3 Prediction models and hyperparameter tuning

The model set was chosen to represent distinct predictive assumptions and levels of model structure rather than to provide an exhaustive comparison of genomic prediction algorithms. rrBLUP/ridge served as a conventional linear-shrinkage baseline with a common L2 penalty. LightGBM represented a nonlinear gradient-boosted tree ensemble capable of modeling piecewise nonlinear effects and interactions. The MLP was intentionally implemented as a standard feed-forward neural network and served as a generic neural comparator capable of learning distributed nonlinear representations; it was not intended to represent a state-of-the-art or heavily specialized deep-learning architecture. In the directed cross-environment analysis, MeNet (Li et al., 2026) was added as a specialized mixed-effects deep neural network developed for multi-environment genomic prediction.

Within each outer training fold, hyperparameters were selected by fivefold inner cross-validation using mean Pearson’s *r* on the inner validation folds. Ridge alpha was searched over {0.1, 1, 10, 100}; alpha = 100 was selected in all final outer folds. LightGBM used eight randomly sampled hyperparameter combinations per outer fold from learning_rate {0.01, 0.03, 0.05, 0.1}, num_leaves {15, 31, 63}, min_child_samples {10, 20, 50}, subsample {0.70, 0.85, 1.00}, colsample_bytree {0.70, 0.85, 1.00}, and n_estimators {100, 160, 240}. The MLP used four randomly sampled hyperparameter combinations per outer fold from hidden_layer_sizes {(64), (128), (128,64), (256,128)}, learning_rate_init {1e-4, 5e-4, 1e-3}, alpha {1e-5, 1e-4, 1e-3}, and batch_size {32, 64, 128}, with ReLU activation, Adam optimization, max_iter = 80, early stopping, and n_iter_no_change = 10. Full search spaces are summarized in **Supplementary Table S4**.

The directed cross-environment analysis used one pre-specified configuration per model across all train–test environment pairs rather than pair-specific tuning. Ridge used alpha = 1.0. LightGBM used learning_rate = 0.05, max_depth = 5, n_estimators = 160, min_child_samples = 20, num_leaves = 10, subsample = 1.0, and colsample_bytree = 1.0. The MLP used hidden layers (128,64), alpha = 1e-4, learning_rate_init = 1e-3, max_iter = 80, early stopping, and n_iter_no_change = 10. For MeNet, 20% of source-environment samples were reserved for validation; batch size was 16, training used 50 epochs with a learning rate of 0.001 and dropout of 0.3 in the VE, RepGeno, and output components. For every directed comparison, model fitting and any validation or early-stopping procedures used only source-environment data; target-environment phenotypes were not used for hyperparameter selection, model fitting, or validation. Fixed settings were retained across directed pairs to provide a common transfer protocol rather than to estimate pair-specific algorithm performance ceilings (**Supplementary Table S4**).

Analyses were implemented in Python using NumPy, pandas, SciPy, scikit-learn, and LightGBM; MeNet used a study-specific adaptation of the official PyTorch codebase. The MeNet architecture contains a variation embedding (VE) branch based on one-dimensional convolution and residual feature extraction, a RepGeno dense branch, feature-fusion layers, and a scalar output head. Exact runtime package versions were not retained in the archived computational environment and are therefore unavailable for retrospective reconstruction. Analysis scripts, configurations, prediction outputs, and figure/table source data are archived in the repository cited in the **Data availability statement**.

### 2.4 Cross-validation and cross-environment evaluation

Within-environment evaluation used five repeats of fivefold outer cross-validation, yielding 25 outer-fold evaluation contexts per model and dataset. Model comparisons were paired within the same dataset, repeat, and fold so that all models were evaluated on the same test individuals. As individuals are reused across repeats, these contexts are correlated resampling contexts rather than independent biological replicates. We therefore use them to summarize empirical performance and fold-to-fold variability, rather than as independent samples for population-level inference.

Cross-environment evaluation used five maize environments labeled BJ, HN, HeB, JL, and LN. Directed train–test pairs were formed by training in one environment and evaluating in another, yielding 20 train ≠ test environment pairs. Test-environment sample sizes ranged from 5,749 to 5,829 hybrids across the five target environments. Directed pairs share both germplasm and environments; multiple pairs reuse the same target environment and individuals. They are therefore correlated evaluation contexts rather than independent environmental replicates, and cross-environment summaries are interpreted descriptively as evidence of contextual dependence.

### 2.5 Prediction equations and response scaling

Let *y* denote the observed phenotype for an individual. The subscript tr denotes quantities estimated from the relevant training data. In the within-environment nested-CV analysis, the standardized response used for model fitting was:

(1) $$z_{i}=\frac{y_{i}-\mu_{\text{tr}}}{\sigma_{\text{tr}}}$$

For Ridge, the standardized-response predictor retained a fitted intercept and L2-regularized marker effects, with marker values standardized from the corresponding training data:

(2) $${\hat{z}}_{i}=b_{0}+\sum_{j=1}^{p}\beta_{j}{\tilde{x}}_{ij}$$

LightGBM generated the prediction for the standardized response using its gradient-boosted tree ensemble:

(3) $${\hat{z}}_{i}=F({\tilde{x}}_{i})$$

The multilayer perceptron generated the prediction for the standardized response using affine neural layers with learned bias terms:

(4) $${\hat{z}}_{i}=f_{\theta }({\tilde{x}}_{i})$$

For all three within-environment models, predictions were returned to the original processed phenotype scale before calculating the reported metrics:

(5) $${\hat{y}}_{i}=\mu_{\text{tr}}+\sigma_{\text{tr}}{\hat{z}}_{i}$$

Equations 1–5 summarize the within-environment response scaling and prediction formulations. In directed cross-environment evaluation, model predictions remained on the source-standardized response scale while observed target-environment EW was independently standardized within the target environment for evaluation. MeNet likewise produced a scalar standardized scale prediction from its genotype and RepGeno representations. No target-phenotype-based *post-hoc* intercept or variance calibration was applied (**Supplementary Table S10**).

Pearson’s *r* and the candidate-level diagnostics used here do not require *post-hoc* target calibration of the prediction vector: *r* is invariant to positive affine transformations, top-k membership and candidate-set overlap depend only on prediction ordering, and OSD@k is calculated from target phenotypes observed in the target environment after the predicted top-k set is identified. Cross-environment RMSE, where reported, is therefore interpreted as a standardized-scale error and not as an error in raw ear-weight units.

### 2.6 Selection performance metrics

For each evaluation context, let n denote the number of test individuals, with observed or adjusted phenotypes and corresponding model predictions defined for the same individuals. We evaluated selection intensities *k* ∈ {5, 10, 20}, where *k* denotes the percentage of candidates retained, to represent stringent, intermediate, and broader candidate-retention budgets. These percentages are operational evaluation levels used to examine sensitivity to selection intensity rather than crop- or program-specific optimal selection intensities.

(6) $$m_{k}=\text{max}(1,\text{ceil}(nk/100))$$

(7) $$T_{k}={\text{Top}}_{m_{k}}(y)$$

(8) $$P_{k}={\text{Top}}_{m_{k}}(\hat{y})$$

Here, T_k_ is the phenotype-defined reference set containing the m_k_ individuals with the largest observed or adjusted phenotype values, whereas *P_k_* is the predicted candidate set containing the m_k_ largest model predictions. *T_k_* is an empirical phenotype-referenced set and should not be interpreted as a set defined by known true breeding values.

(9) $$\text{Recovery}@k=\frac{\left|T_{k}\cap P_{k}\right)}{m_{k}}$$

(10) $$\text{OSD}@k=\frac{{\text{mean}}_{i\in P_{k}}(y_{i})-\text{mean}(y)}{{\text{SD}}_{\text{pop}}(y)}$$

Observed top-k recovery quantifies the fraction of phenotype-defined reference candidates recovered by the predicted set. OSD@k expresses the observed phenotypic advantage of the predicted candidate set in standard-deviation units of the evaluation population. It is a retrospective phenotype-referenced diagnostic and is not an estimate of realized genetic gain or response to selection in a subsequent generation.

(11) $${\text{Overlap}}_{k}(A,B)=\frac{\left|P_{k}^{A}\cap P_{k}^{B}\right)}{m_{k}}$$

(12) $$J_{k}(A,B)=\frac{\left|P_{k}^{A}\cap P_{k}^{B}\right)}{\left|P_{k}^{A}\cup P_{k}^{B}\right)}$$

(13a) $$TP=|T_{k}\cap P_{k}),FP=|P_{k})-TP.$$

(13b) $$FN=|T_{k})-TP,TN=n-TP-FP-FN.$$

Equations 6–13b define the selection metrics and confusion-matrix quantities used here. Sensitivity/recall, specificity, precision, and F1 were calculated from these counts using their standard definitions. As both T_k_ and P_k_ contain exactly m_k_ individuals, FP = FN and therefore precision = sensitivity/recall = F1 = Recovery@k. These quantities are reported for interpretability rather than as independent performance evidence (**Supplementary Table S9**).

Candidate sets were obtained by sorting the relevant phenotype or prediction vector in descending order and retaining exactly the first m_k_ positions. Ties at the cutoff were not expanded beyond m_k_. In the archived implementation, any exact cutoff tie was therefore resolved by the sorting order applied to the fixed input array. An audit of archived prediction-level files found no model prediction ties at the evaluated top-k boundaries; phenotype cutoff ties were uncommon and involved only small tied groups.

### 2.7 Decision-level analyses

We performed three decision-level analyses. First, model choice reversal assessed whether the model with the highest Pearson’s *r* in an evaluation context was the same as the model with the highest observed top-k recovery or OSD@k. We call these nominal reversals because a reversal can occur when the performance difference between competing models is very small. Reversal rate is therefore used as a screening summary of decision ambiguity rather than as evidence of realized downstream genetic response or loss.

Second, pairwise *r*-selection discordance compared every model pair within each evaluation context. We defined near-*r* comparisons operationally as |Δ*r*| ≤ 0.02, strict recovery discordance as a near-*r* plus |ΔRecovery| ≥ 0.08, and strict OSD discordance as near-*r* plus |ΔOSD| ≥ 0.15. Because these are conditional descriptive events, we report raw numerators, denominators, and percentages without binomial confidence intervals. Sensitivity analyses varied the near-*r* threshold (0.01, 0.02, 0.05), recovery threshold (0.05, 0.08, 0.10), and OSD threshold (0.10, 0.15, 0.20) to assess dependence on the operational cutoffs (**Supplementary Table S8**).

Third, top-k overlap analysis asked whether models with similar *r* prioritized the same candidate materials. This analysis focused on predicted top-5%, top-10%, and top-20% sets and does not by itself determine which model prioritized better candidates. For representative near-*r* cases, we additionally examined candidate turnover and, where synchronized prediction-level outputs were available, whole-sample versus upper-tail rank concordance (**Supplementary Tables S7**, **S11**).

## 3 Results

### 3.1 Within-environment model performance showed a clear model-tier pattern

The within-environment analyses used the processed maize NCII6210 EW dataset and the switchgrass G3 HT dataset (**Table 1**). In maize, rrBLUP/ridge and LightGBM showed very similar predictive ability, whereas MLP was lower. Mean Pearson’s *r* was 0.803 ± 0.009 for rrBLUP/ridge, 0.796 ± 0.009 for LightGBM, and 0.749 ± 0.020 for MLP. At k = 5%, observed top-k recovery was 0.479 ± 0.049, 0.468 ± 0.046, and 0.416 ± 0.063, respectively; OSD@5 was 1.588 ± 0.088, 1.568 ± 0.087, and 1.463 ± 0.113 (**Figure 2**; **Supplementary Table S1**). Values are mean ± SD across the 25 correlated outer-fold evaluation contexts and describe empirical fold-to-fold dispersion rather than inferential uncertainty.

**Figure 2:**
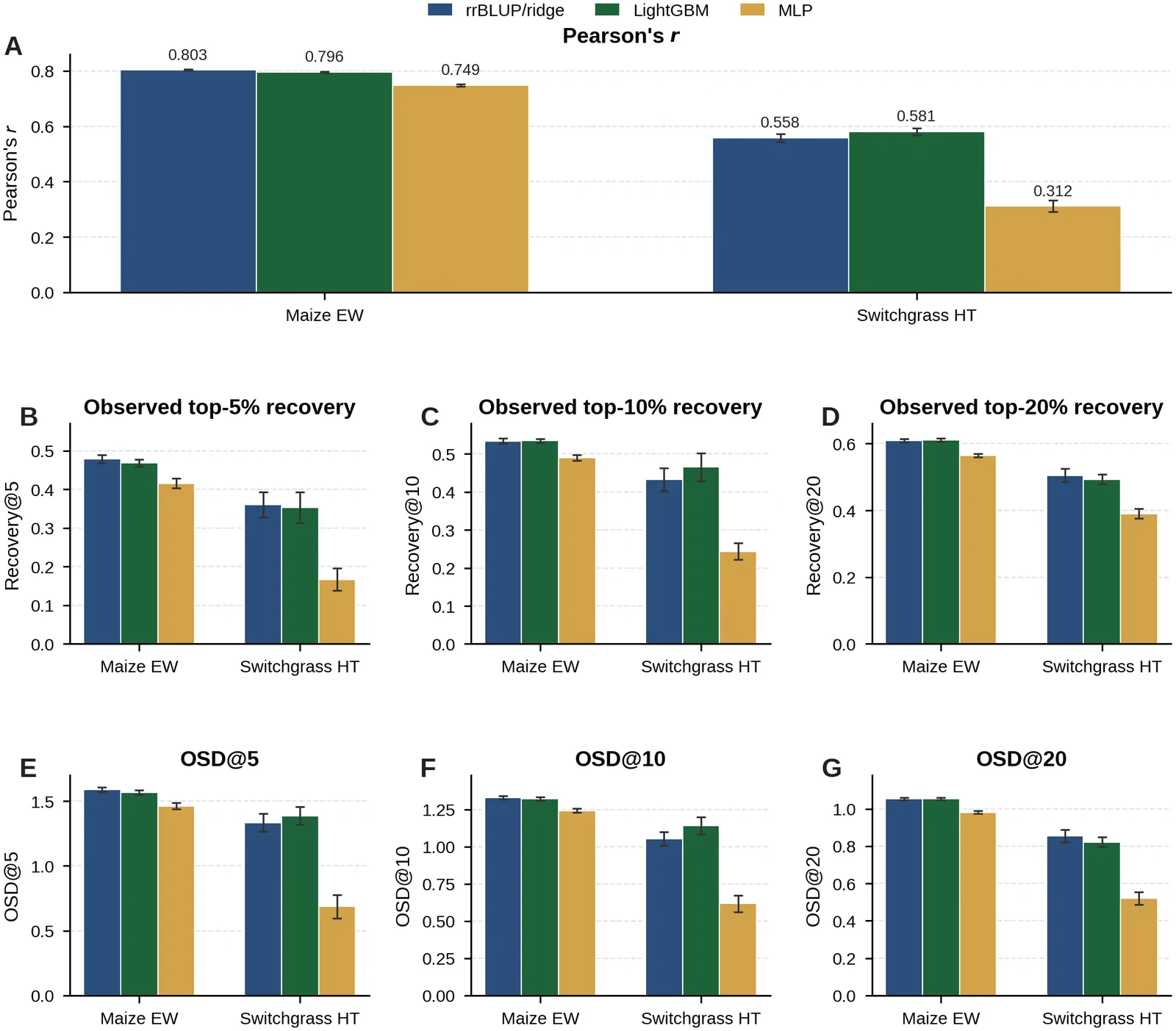
Within-environment model performance across 25 outer-fold evaluation contexts. **(A)** Pearson’s *r*. **(B–D)** Observed top-k recovery at *k* = 5%, 10%, and 20%. **(E–G)** Standardized observed selection differential at *k* = 5%, 10%, and 20%. Error bars show SD across the 25 outer-fold contexts (5 repeats × 5 folds) and display empirical fold-to-fold variability rather than inferential uncertainty for rrBLUP/ridge, LightGBM, and MLP in maize NCII6210 EW and switchgrass G3 HT.

Switchgrass was a more difficult prediction task. Mean Pearson’s *r* was 0.581 ± 0.060 for LightGBM, 0.558 ± 0.071 for rrBLUP/ridge, and 0.312 ± 0.106 for MLP. Recovery@5 was 0.360 ± 0.164 for rrBLUP/ridge, 0.353 ± 0.200 for LightGBM, and 0.167 ± 0.144 for MLP. This pattern supports the model-tier interpretation: clearly weaker model implementations tended to be weaker across both global and phenotype-referenced candidate diagnostics, whereas rrBLUP/ridge and LightGBM were close enough that decision-level comparisons varied by fold or dataset (**Figure 2**; **Supplementary Table S1**).

Values are mean ± SD across 25 outer-fold evaluation contexts (5 repeats × 5 folds). SD describes empirical fold-to-fold dispersion only; the contexts are correlated resampling evaluations and are not independent biological replicates.

### 3.2 Cross-environment prediction retained the broad model-tier pattern

The cross-environment maize EW analysis compared rrBLUP, LightGBM, MLP, and MeNet across 20 directed train-test environment pairs. Mean Pearson’s *r* was 0.408 for LightGBM, 0.402 for rrBLUP, 0.396 for MeNet, and 0.345 for MLP. At *k* = 5%, mean observed top-k recovery was 0.202, 0.193, 0.183, and 0.173, respectively, and mean OSD@5 was 0.754, 0.748, 0.729, and 0.701. The distributions of Pearson’s *r*, observed top-20% recovery, and OSD@20 across the 20 environment pairs are shown in **Figure 3**. Mean values for all three selection intensities are provided in **Supplementary Table S5**.

**Figure 3:**
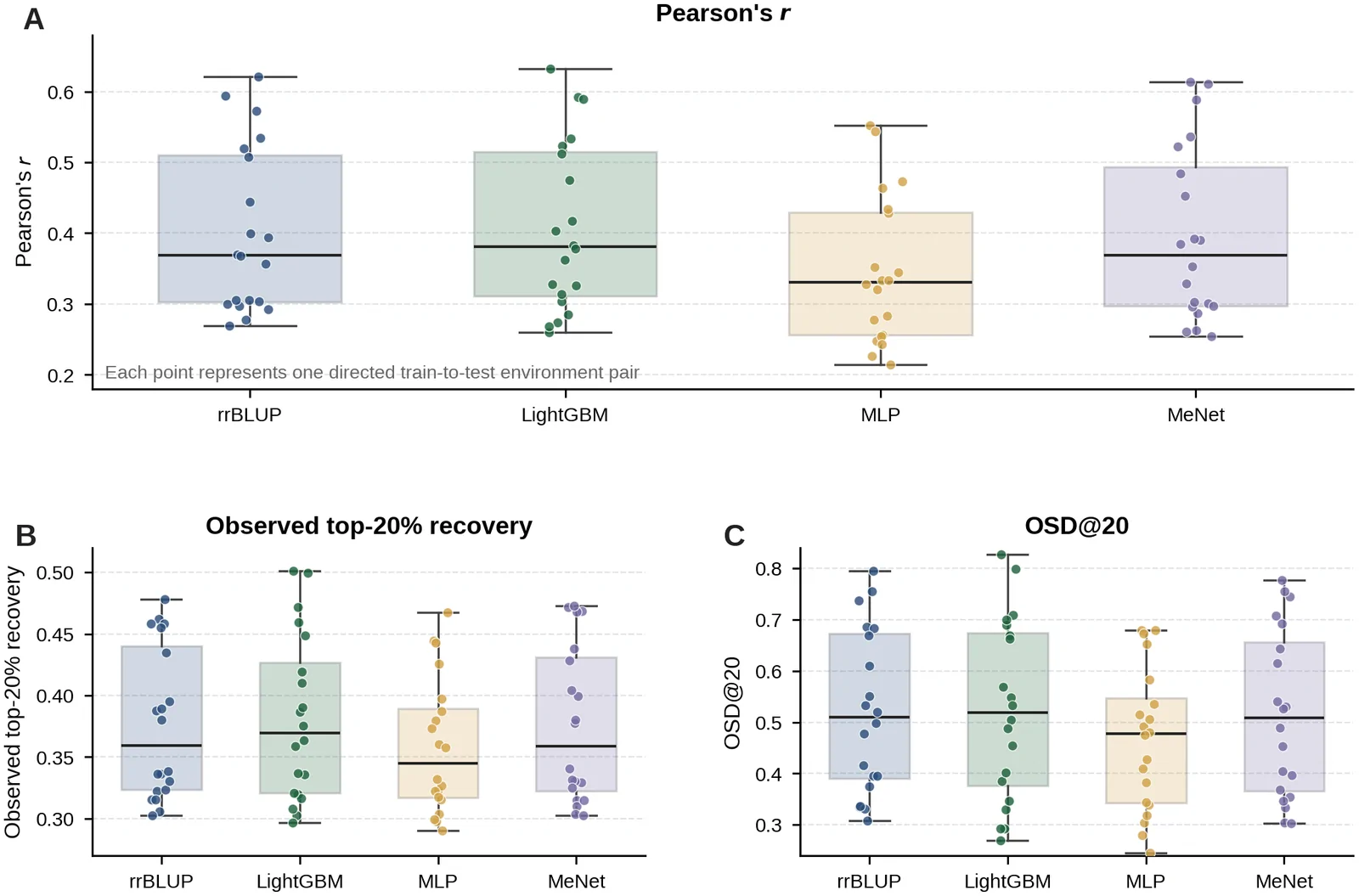
Cross-environment prediction performance across 20 directed train–test environment pairs in maize. Models were trained in one of five environments (BJ, HN, HeB, JL, or LN) and evaluated in a different environment. Each point represents an exact directed environment-pair result from the final analysis table. Boxplots show the median, interquartile range, and 1.5 × IQR whiskers. **(A)** Pearson’s *r*. **(B)** Observed top-20% recovery. **(C)** OSD@20. The comparison includes rrBLUP, LightGBM, MLP, and MeNet.

The cross-environment distributions retained the same broad hierarchy observed within environments: LightGBM, rrBLUP, and MeNet largely overlapped, whereas the evaluated MLP implementation tended to be lower. Nevertheless, pair-specific variation was substantial, and nominal model choice disagreement remained common: depending on k and the candidate metric, the *r*-best model differed from the recovery-best or OSD-best model in 25%–55% of directed environment pairs. These pair-level summaries are descriptive because directed pairs share environments and germplasm (**Figure 3**; **Supplementary Table S5**).

### 3.3 The *r*-best model was not always the selection-best model

We next asked whether the model with the highest Pearson’s *r* in a given evaluation context was also the model with the highest observed top-k recovery or OSD@k. Within the two within-environment tasks, nominal disagreement between the *r*-best model and the recovery-best model occurred in 40.0%, 42.0%, and 48.0% of contexts at *k* = 5%, 10%, and 20%, respectively. Nominal disagreement between the *r*-best model and the OSD-best model occurred in 34.0%, 32.0%, and 54.0% of contexts. In the maize cross-environment task, *r*-versus-recovery disagreement occurred in 45.0%, 55.0%, and 25.0% of contexts, and *r*-versus-OSD disagreement occurred in 45.0%, 45.0%, and 35.0% of contexts (**Figure 4**; **Supplementary Table S2**).

**Figure 4:**
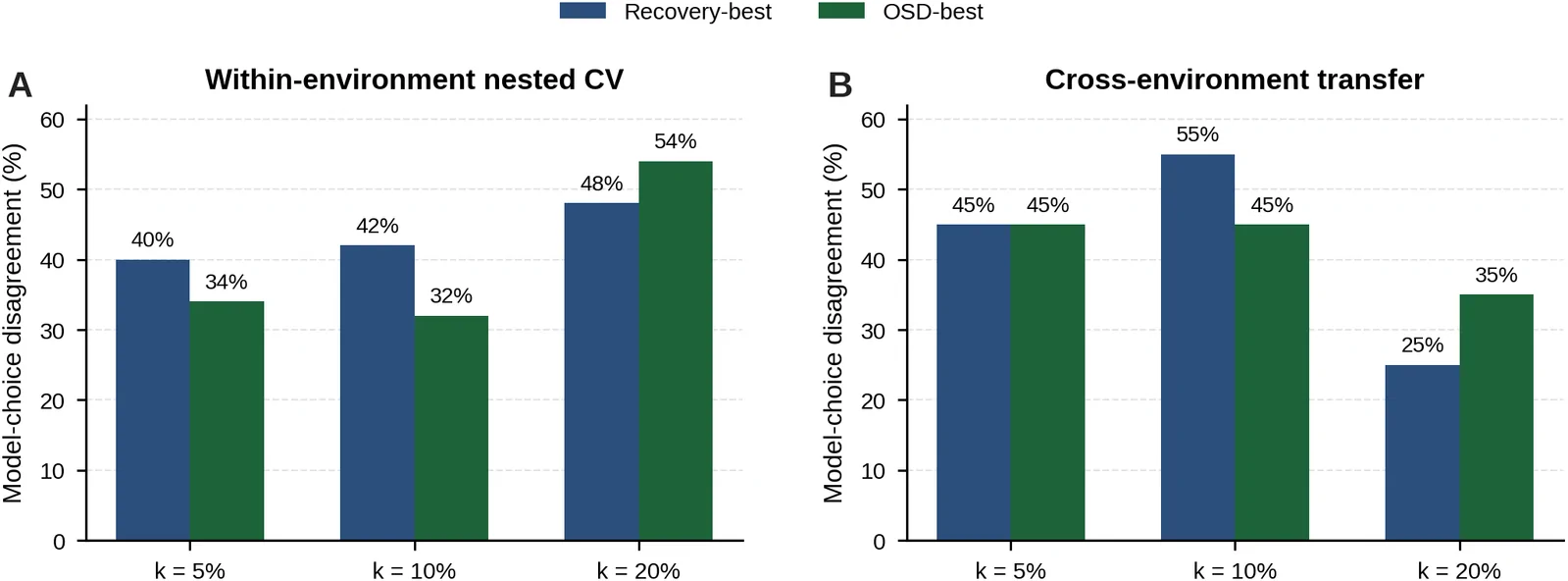
Model selection reversal between Pearson’s *r* and phenotype-referenced candidate metrics. **(A)** Within-environment nested-CV results. **(B)** Cross-environment transfer results. Bars show the proportion of evaluation contexts in which the model with the highest Pearson’s *r* differed from the model with the highest observed top-k recovery or OSD@k. Percentages are descriptive only because evaluation contexts are correlated.

These reversal rates show that different metrics can name different best models, but they should not be overinterpreted. A nominal reversal can be driven by very small differences between nearly tied models. Reversal rate is therefore useful as a screening statistic for decision ambiguity, but it does not quantify realized genetic loss from choosing a model by *r*. In the fixed-cardinality top-k formulation, precision, recall, and F1 are mathematically identical to Recovery@k; the added confusion-matrix value is the explicit decomposition into TP, FP, FN, TN, and specificity rather than three independent performance measures (**Supplementary Table S9**).

### 3.4 Strict near-*r* discordance was present but not frequent

To focus on the biologically relevant question of whether very similar *r* values can mask meaningful selection differences, we restricted the denominator to near-*r* model pairs with |Δ*r*| ≤ 0.02. In the within-environment analysis, we identified 33 near-*r* pairwise comparisons at each *k*. Strict recovery discordance occurred in three of 33 comparisons at *k* = 5%, 5/33 at *k* = 10%, and 5/33 at *k* = 20%, corresponding to conditional rates of 9.1%, 15.2%, and 15.2%. Strict OSD discordance occurred in five of 33, five of 33, and four of 33 comparisons, corresponding to 15.2%, 15.2%, and 12.1%.

In the cross-environment analysis, we identified 48 near-*r* pairwise comparisons at each *k*. Strict recovery discordance occurred in one of 48 comparisons at *k* = 5% and in none of the comparisons at *k* = 10% or *k* = 20%. Strict OSD discordance occurred in four of 48 comparisons at *k* = 5%, one of 48 at *k* = 10%, and none at *k* = 20%. These results support a restrained interpretation. Strong *r*-selection discordance can occur, but it was not the dominant pattern in the final data considered here (**Figure 5**; **Supplementary Table S2**).

**Figure 5:**
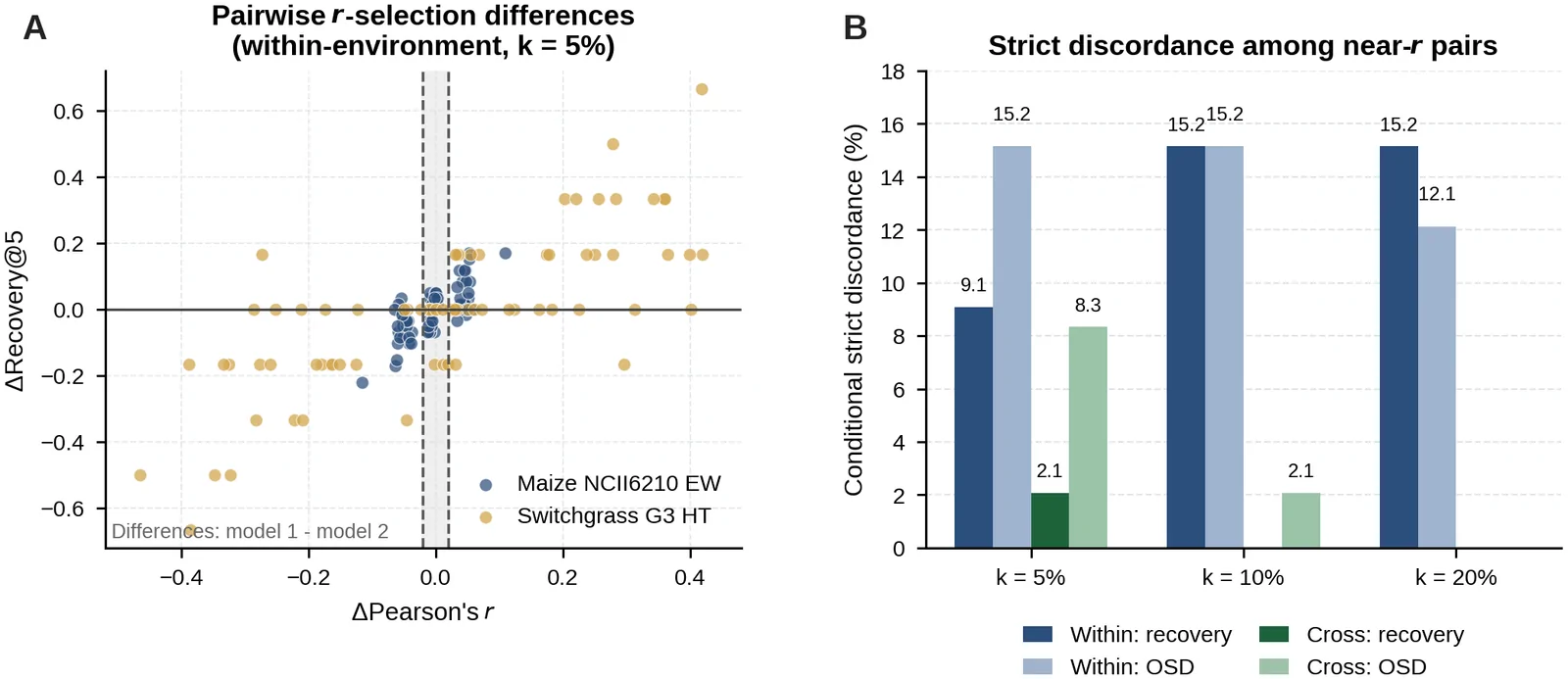
Pairwise *r*-selection discordance. **(A)** Exact fold-level relationship between differences in Pearson’s *r* and observed top-5% recovery across all within-environment model pair comparisons; the colors distinguish maize NCII6210 EW and switchgrass G3 HT, and dashed vertical lines mark the near-*r* region |Δ*r*| ≤ 0.02. **(B)** Conditional strict recovery and OSD discordance rates among near-*r* comparisons at *k* = 5%, 10%, and 20% in within- and cross-environment evaluations.

Sensitivity analyses confirmed that the exact conditional percentages changed as the operational near-*r*, recovery, and OSD cutoffs were tightened or relaxed, but the qualitative interpretation was stable: strong near-*r* discordance remained a minority pattern and was rarer in the directed cross-environment comparisons than nominal reversal rates alone suggested (**Supplementary Table S8**). The cutoffs are therefore used as operational criteria rather than as universal biological boundaries.

### 3.5 Representative discordance cases revealed two decision-level pathways

Representative cases clarified how near-equal global predictive ability could produce different candidate-level outcomes. In switchgrass repeat 1, fold 4 at *k* = 5%, LightGBM and rrBLUP/ridge differed by only |Δ*r*| = 0.0006 and had identical observed top-5% recovery (0.333 for both), yet their top-5% sets overlapped by only 66.7% and OSD@5 differed by 0.628. Thus, discordance can arise even when the same number of phenotype-defined top candidates is recovered, because the nonshared candidates differ in observed phenotypic performance.

A second switchgrass case showed both recovery and composition differences. In repeat 3, fold 4 at *k* = 5%, LightGBM had a slightly higher *r* than rrBLUP/ridge (0.668 vs. 0.656), but rrBLUP/ridge had higher observed top-5% recovery (0.500 vs. 0.333) and higher OSD@5 (1.801 vs. 1.422). The predicted top-5% sets overlapped by only 50.0%. This case illustrates that a modest global-*r* advantage does not necessarily determine which model performs better under phenotype-referenced candidate diagnostics.

The same mechanism was evident under directed cross-environment transfer. For HeB→BJ at *k* = 5%, LightGBM and rrBLUP differed by |Δ*r*| = 0.0109. Their predicted scores remained strongly rank-correlated across the full target population (Spearman *ρ* = 0.871), but their top-5% sets overlapped by only 55.0%, and rank concordance within the union of selected candidates decreased to *ρ* = 0.098. Of 291 selected positions per model, 131 were not shared. LightGBM-only candidates had a mean target-standardized observed phenotype of 1.185, compared with 0.839 for rrBLUP-only candidates, consistent with the observed OSD@5 difference (1.243 vs. 1.088). These cases identify upper-tail candidate turnover and local rank rearrangement as proximate decision-level sources of discordance (**Supplementary Tables S7**, **S11**).

### 3.6 Similar-*r* models selected partially different top-ranked candidates

The aggregate overlap analysis provided a broader view of candidate identity differences. In maize, LightGBM and rrBLUP/ridge had very similar *r* across folds (mean |Δ*r*| = 0.0077), but their predicted top-5% sets had a mean overlap of 72.9%, increasing to 76.6% at top-10% and 81.6% at top-20%. Thus, even when the two stronger models were close in global predictive ability, approximately one-quarter of the top-5% candidate identities differed on average (**Figure 6**; **Supplementary Table S3**).

**Figure 6:**
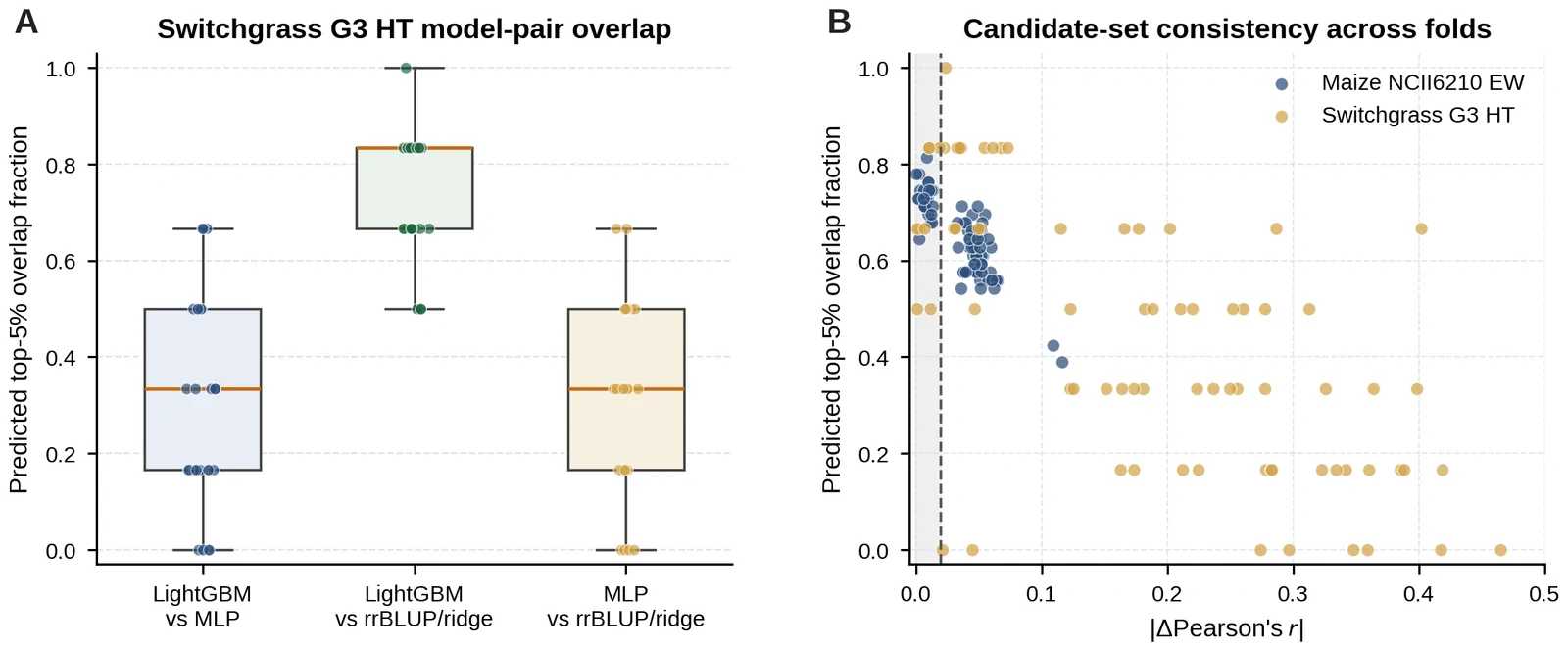
Candidate set consistency across model comparisons. **(A)** Exact predicted top-5% overlap across 25 folds for each model pair in switchgrass G3 HT. **(B)** Relationship between |ΔPearson’s *r*| and exact predicted top-5% overlap across maize NCII6210 EW and switchgrass G3 HT. Similar global predictive performance did not necessarily result in identical candidate decisions.

Switchgrass showed larger candidate identity differences in some folds. For near-*r* LightGBM versus rrBLUP/ridge comparisons, mean overlap was 68.8%, 78.4%, and 68.5% at top-5%, top-10%, and top-20%, respectively; corresponding minimum overlaps were 50.0%, 54.5%, and 57.1%. Top-k overlap should not be interpreted as identifying the better model; it answers the distinct operational question of whether similar-*r* models would prioritize the same materials (**Figure 6**; **Supplementary Table S3**).

Overlap values are percentages of the selected top-k set shared between two models. Fold-level overlap summaries are descriptive because repeated cross-validation contexts are correlated.

## 4 Discussion

### 4.1 Global predictive ability and selection-oriented performance are related but distinct

Previous studies have reached different conclusions about how closely global correlation tracks selection-stage performance, and our results help reconcile those views. Ornella et al. (2014) compared Pearson’s *r* with Cohen’s kappa and a relative-efficiency measure for selecting extreme fractions in maize and wheat and found these quantities to be closely related for most traits at 15% selection. That result is consistent with our observation that clearly weaker model implementations were generally also weaker under phenotype-referenced candidate diagnostics and that strict discordance among near-*r* pairs was a minority pattern. By contrast, Blondel et al. (2015) reported poor agreement between Pearson’s correlation and NDCG across six genomic-selection datasets and showed that models with strong global correlation were not necessarily strongest for top-ranked individuals. Our results do not contradict that finding; rather, they narrow the question to model pairs that are already close in *r* and show that meaningful upper-tail disagreement can still occur, although it is not the dominant outcome of every near-*r* comparison.

More recent work further emphasizes that model evaluation can depend on the selection decision itself. Heinrich et al. (2025) showed with normalized cumulative gain that relative model performance can change with the number of selected individuals, while Montesinos-Lopez et al. (2025) optimized classification thresholds to balance sensitivity and specificity for identifying top or bottom lines. Our results confirm the practical importance of selection-focused evaluation but extend this literature in two specific ways: we condition comparisons on near-equal Pearson’s *r* to distinguish nominal model-choice reversals from large decision differences, and we quantify candidate-set identity separately from phenotype-referenced performance. These distinctions matter because two models can achieve similar Recovery@k while advancing different nonshared candidates, or can have similar global predictive ability while disagreeing locally in the selected tail.

Accordingly, the novelty of the present framework is not a new ranking statistic or a claim that Pearson’s *r* is generally inadequate. It is the conditional integration of global predictive ability, strict near-*r* discordance, candidate identity, and directed cross-environment transfer within the same evaluation contexts. This positions candidate diagnostics as a targeted second-stage audit when models are difficult to distinguish globally, rather than as universal replacements for conventional measures of predictive ability.

From a breeding-cycle perspective, the practical consequence of near-*r* discordance is that two models with similar population-wide predictive ability can advance different subsets of lines. If the non-shared candidates differ in additive genetic merit, those choices could lead to different realized responses in a subsequent breeding cycle even when Pearson’s *r* is nearly unchanged. In our analyses, OSD@k provides a retrospective bridge between candidate composition and selection-stage phenotypic performance by summarizing the observed phenotypic advantage of the model-selected set. It should not, however, be interpreted as realized genetic gain, because realized response also depends on phenotype reliability, the genetic component of the observed differences, relatedness and mating design, and descendant performance. Prospective multicycle or independent-progeny validation would therefore be required to quantify the genetic consequences of choosing one near-*r* model over another.

### 4.2 Near-*r* discordance arose from upper-tail candidate turnover

The representative cases identify two proximate decision-level pathways. Recovery discordance occurs when candidate turnover changes how many phenotype-defined top candidates are recovered. Composition discordance can occur even when Recovery@k is identical, because the non-shared selected candidates can differ in observed phenotype and therefore in their contribution to OSD@k. The HeB→BJ transfer case further showed that the models could remain strongly rank-correlated across the full population while concordance decreased sharply in the selected upper tail. Thus, near-*r* discordance reflected local rank rearrangement around the selection boundary rather than wholesale disagreement across the full prediction distribution.

This case-level decomposition extends earlier ranking, coincidence/selected-set agreement, and top-fraction studies because most existing selection-oriented summaries reduce candidate behavior to a single score such as kappa, relative efficiency, NDCG, normalized cumulative gain, or sensitivity/specificity. Those metrics are useful for asking whether a model selects high-performing individuals, but they do not by themselves identify why two near-*r* models disagree in a specific evaluation context. Here, the switchgrass case with identical Recovery@5 but a large OSD difference shows that equal recovery does not imply the same replacement candidates, while the HeB→BJ case shows that high whole-population rank concordance can coexist with weak upper-tail concordance. Candidate identity is therefore an additional decision dimension rather than merely another transformation of the ranking score.

The evaluated algorithms impose different inductive biases that provide a plausible mechanism for this local reordering. rrBLUP/ridge aggregates marker information through a single L2-regularized linear predictor, whereas LightGBM constructs local threshold rules and interactions through boosted trees; the neural models distribute signal through nonlinear hidden representations. Blondel et al. (2015) found tree-based ensemble methods to be strong performers for ranking in their multidataset comparison, but our results do not indicate a universal LightGBM advantage over linear shrinkage. The direction and magnitude of near-*r* discordance varied by evaluation context, reinforcing that average ranking performance for a model class and pair-specific decision consistency are distinct questions. The present analysis also does not establish a locus-level genomic mechanism; direct comparison of Ridge coefficients, LightGBM importance scores, and neural network attributions would require a harmonized cross-model attribution design, and specific SNPs or genomic regions cannot be inferred from the present results.

### 4.3 Directed cross-environment evaluation adds a transfer-decision perspective

Most multienvironment genomic prediction studies have focused on improving predictive ability by modeling genotype-by-environment structure, environment-specific effects, or nonlinear genomic representations. Crossa et al. (2025) reviewed the growing role of statistical machine learning and deep learning in expanding genomic prediction, while Li et al. (2026) developed MeNet specifically for multi-environment prediction and reported superior predictive performance in 34 of 36 evaluations across rice, wheat, and maize. That literature establishes the importance of environment-aware model development, but its primary focus is usually the comparison of algorithmic predictive performance. Our directed transfer experiment asks a different, complementary question: when a model is trained in one environment and transferred to another, does similar global transfer performance imply that the same candidates would be prioritized?

Under the common source-only transfer protocol used here, LightGBM, rrBLUP, and MeNet showed broadly overlapping distributions of global and candidate-level metrics, whereas the evaluated MLP implementation was generally lower. This result should not be read as contradicting the stronger benchmark performance reported for MeNet by Li et al. (2026), because our analysis is not a replication of their multienvironment training and tuning design. We used fixed configurations across directed pairs, no target-environment phenotypes for tuning or validation, and a train-one-environment/test-one-environment transfer setting. These choices were intended to create a common transfer protocol for studying metric and candidate behavior, not to estimate pair-specific performance ceilings for the competing algorithms.

The extension contributed by our cross-environment analysis is therefore decision consistency rather than a new claim about which model class predicts best across environments. In the HeB→BJ example, the competing models differed by only 0.0109 in Pearson’s *r* and remained strongly concordant across the full target population (Spearman’s rho ≈ 0.871), yet upper-tail rank concordance was weak (rho ≈ 0.098), and only about 55% of the top-5% candidates were shared. This shows that transfer accuracy and selection stability are related but non-equivalent properties. For breeding programs that advance a fixed quota of lines into a target environment, a candidate-overlap audit can therefore be informative when global transfer performance is nearly tied. The present data, however, represent environmental heterogeneity rather than stress-specific climate validation.

### 4.4 Phenotype-referenced candidate diagnostics complement bias, calibration, and reliability information

Observed top-k recovery and OSD@k are retrospective phenotype-referenced diagnostics. The reference set is defined by observed or adjusted phenotypes rather than known true breeding values, and those phenotypes contain environmental and measurement components. Consequently, these metrics do not demonstrate superior recovery of true breeding values, realized genetic gain, or response to selection in a subsequent generation. They address a narrower operational question: given the available phenotype reference in the evaluation population, which candidates would different models prioritize, and how those candidates performed phenotypically?

Source study reliability information reinforces this distinction. Lipka et al. (2014) reported a clone-mean repeatability of 0.75 (SE = 0.03) for switchgrass plant height, but the benchmark used here was subsequently reformatted and normalized, and the final processed sample intersection differs from the source reliability sample. For maize hybrid EW, the source used multienvironment BLUP processing but did not provide a directly matching numerical reliability estimate for the processed F1 response analyzed here. We therefore do not transform phenotype-prediction correlations into genetic value accuracy estimates. This candidate-focused perspective also complements, rather than replaces, established attention to prediction bias and dispersion/calibration (Legarra and Reverter, 2018; Gowane et al., 2019).

The confusion-matrix formulation provides an additional interpretive lens but does not provide several independent outcomes. As the phenotype-defined and predicted top-k sets have the same cardinality, FP = FN and precision = recall = F1 = Recovery@k by construction. Its added value is therefore the explicit decomposition of omitted and promoted candidates and specificity, not multiple independent pieces of evidence.

### 4.5 Practical implications and limitations

The practical value of the proposed framework lies in triage rather than metric proliferation. If competing models are clearly separated in Pearson’s *r* and occupy the same broad performance tier under candidate diagnostics, additional selection summaries are unlikely to change the operational conclusion. When models are closely matched, however, observed top-k recovery and OSD@k describe the phenotype profile of prioritized candidates, while candidate-set overlap indicates whether the same materials would actually be advanced. A three-stage workflow is therefore appropriate: report *r* and error metrics routinely; add phenotype-referenced candidate diagnostics when *r* differences are small or selection intensity is high; and inspect candidate overlap and case-level upper-tail ordering when candidate identity itself is consequential. This conditional workflow is the main practical novelty of the study: it identifies when existing selection-oriented metrics provide actionable information beyond global predictive ability without requiring breeders to replace *r* as a routine first-line summary.

Several limitations constrain the scope of inference. The study uses two within-environment tasks and one maize five-location cross-environment task rather than a broad multicrop benchmark. Repeated outer-fold contexts reuse individuals across repeats, and directed environment pairs share both environments and germplasm; they are correlated evaluation contexts rather than independent biological replicates. Accordingly, fold- and pair-level proportions are interpreted descriptively, without binomial confidence intervals or population-level inference. The near-*r* and strict discordance cutoffs are operational criteria, although sensitivity analyses showed that the qualitative interpretation was stable across reasonable alternatives.

The present datasets were not designed as explicit drought, heat, salinity, or other abiotic-stress trials. They provide contrasting within- and cross-environment case studies of environmental heterogeneity, but the results should not be interpreted as direct evidence of improved performance under specific climate stresses or of climate resilience. Finally, the analyses are retrospective comparisons of metrics on a common set of fitted predictions; they do not test whether choosing a model by Recovery@k or OSD@k in an inner selection stage improves future held-out performance. A stronger future design would use nested metric-driven model selection and quantify the resulting selection regret in an independent outer evaluation.

## 5 Conclusion

The contribution of this study is not a new ranking or selection metric, but a conditional evaluation framework that links global predictive ability to phenotype-referenced candidate behavior. Across the maize and switchgrass case studies, Pearson’s *r* remained informative for broad model evaluation, while near-*r* discordance and candidate set analyses identified the smaller subset of comparisons in which similar global performance did not imply the same candidate selection outcome. Extending this framework to directed cross-environment prediction further showed how candidate-level consistency can be evaluated alongside overall transfer performance. Observed top-k recovery, OSD@k, and predicted top-k overlap are therefore best viewed as targeted retrospective complements to established predictive ability, error, bias, calibration, and ranking summaries. As these diagnostics are evaluated against observed or adjusted phenotypes, they do not by themselves demonstrate the recovery of the highest true breeding values, realized genetic gain, or future response to selection. Such complementary diagnostics may support more transparent and stable genomic-selection decisions across heterogeneous environments and may be useful in multienvironment breeding programs.

## Data Availability

The fold-level prediction results, source data underlying the reported figures and tables, and analysis scripts are publicly available in Zenodo at https://doi.org/10.5281/zenodo.21703284. Publicly available datasets were analyzed in this study. This data can be found here: The switchgrass benchmark genotype and phenotype data are available in the Dryad repository at https://doi.org/10.5061/dryad.xksn02vb9. The maize NCII6210 genotype and phenotype data are available through the ZEAMAP/MaizeGo CUBIC-related data repository at http://zeamap.hzau.edu.cn/ftp/99_MaizegoResources/01_CUBIC_related/. The associated source article is available at https://doi.org/10.1186/s13059-021-02370-7.
