# Supplementary material for: Selection-aware metrics provide complementary decision-level information in plant genomic prediction
Source: Front Plant Sci. 2026 Oct 1;17:1951182. doi: 10.3389/fpls.2026.1951182 (PMC13668517; doi:10.3389/fpls.2026.1951182)
Supplement: Supplementary file 1 [file Supplementaryfile1.docx]

**Supplementary Information**

**Selection-aware metrics provide complementary decision-level information in plant genomic prediction**

Tables S1–S11

**Supplementary Results**

**S1. Upper-tail rank rearrangement and candidate turnover**

Among near-*r* comparisons, strong decision-level discordance was a minority pattern, but representative cases showed that it could arise through more than one pathway. In switchgrass, strict cases generally showed lower candidate set overlap than non-strict near-*r* cases, particularly at higher selection intensity. Case-level decomposition further showed that OSD discordance could occur even when observed top-k recovery was identical, because the non-shared candidates differed in observed phenotype. Under directed cross-environment transfer, competing near-*r* models could remain strongly rank-correlated across the full target population while becoming weakly concordant in the selected upper tail. These results support candidate turnover and local upper-tail rank rearrangement as the immediate decision-level source of the observed discordance (Supplementary Tables S7 and S11).

**S2. Sensitivity to operational discordance thresholds**

The exact conditional discordance percentages varied when the near-*r* and strict-difference cutoffs were tightened or relaxed, as expected for operational thresholds. Across the examined settings, however, strong discordance remained a minority pattern rather than the predominant behavior of near-*r* model pairs. Cross-environment strict recovery discordance remained especially uncommon, whereas OSD discordance was somewhat more frequent at k = 5%. These results support reporting the primary cutoffs as descriptive operational filters rather than universal biological or breeding-decision boundaries (Supplementary Table S8).

**S3. Confusion-matrix interpretation of fixed-budget top-k selection**

Recasting the observed and predicted top-k sets as positive classes made candidate-selection errors explicit in terms of TP, FP, FN, and TN. Because the phenotype-defined and predicted sets contain exactly the same number of individuals, FP = FN and precision, sensitivity/recall, F1, and observed top-k recovery are mathematically identical. The additional value of this analysis is therefore interpretive: it shows the absolute numbers of phenotype-defined top candidates recovered, missed, or replaced and provides specificity as a complementary class-imbalance diagnostic. Dispersion is reported as SD across the correlated evaluation contexts and is descriptive rather than inferential (Supplementary Table S9).

**S4. Prediction scale, tie handling, and implementation detail**

Supplementary Table S10 summarizes prediction equations, intercept/bias terms, genotype scaling, response standardization, and evaluation scales for all model/setting combinations. Candidate sets retain the fixed cardinality defined in Methods Section 2.6; exact prediction-score ties at the evaluated top-k boundaries were not detected in the archived prediction-level audit, while phenotype cutoff ties were uncommon and involved small tied groups. Exact runtime package versions were not reconstructed retrospectively because the archived execution environments did not retain a verifiable version snapshot.

**Supplementary Tables**

Supplementary Table S1. Within-environment model performance summary across 25 outer-fold evaluation contexts.

Supplementary Table S2. Model choice reversal and conditional near-*r* discordance summary.

Supplementary Table S3. Predicted top-k overlap across model pairs, datasets, and selection intensities.

Supplementary Table S4. Model implementation and hyperparameter settings.

Supplementary Table S5. Cross-environment metrics by selection intensity and model.

Supplementary Table S6. Marker quality-control summary for the processed input matrices.

Supplementary Table S7. Near-*r* discordance mechanism examples.

Supplementary Table S8. Sensitivity of strict discordance rates to operational thresholds.

Supplementary Table S9. Selection confusion-matrix metrics under fixed-cardinality top-k selection.

Supplementary Table S10. Prediction equations, intercept/bias terms, and scaling by model and evaluation setting.

Supplementary Table S11. Representative near-*r* strict discordance cases and decision-level decomposition.

**Supplementary Table S1. Within-environment model performance summary across 25 outer-fold evaluation contexts.**

| **Dataset** | **Model** | **Pearson *r*** | **RMSE** | **Recovery@5** | **OSD@5** | **Recovery@10** | **OSD@10** | **Recovery@20** | **OSD@20** |
| --- | --- | --- | --- | --- | --- | --- | --- | --- | --- |
| Maize NCII6210 EW | LightGBM | 0.796 ± 0.009 | 12.050 ± 0.178 | 0.468 ± 0.046 | 1.568 ± 0.087 | 0.535 ± 0.027 | 1.322 ± 0.051 | 0.611 ± 0.028 | 1.054 ± 0.031 |
| Maize NCII6210 EW | MLP | 0.749 ± 0.020 | 13.552 ± 0.516 | 0.416 ± 0.063 | 1.463 ± 0.113 | 0.490 ± 0.036 | 1.242 ± 0.066 | 0.565 ± 0.025 | 0.982 ± 0.035 |
| Maize NCII6210 EW | rrBLUP/ridge | 0.803 ± 0.009 | 11.849 ± 0.207 | 0.479 ± 0.049 | 1.588 ± 0.088 | 0.534 ± 0.033 | 1.329 ± 0.057 | 0.610 ± 0.025 | 1.053 ± 0.031 |
| Switchgrass G3 HT | LightGBM | 0.581 ± 0.060 | 0.815 ± 0.062 | 0.353 ± 0.200 | 1.386 ± 0.343 | 0.465 ± 0.182 | 1.142 ± 0.293 | 0.493 ± 0.070 | 0.822 ± 0.134 |
| Switchgrass G3 HT | MLP | 0.312 ± 0.106 | 1.312 ± 0.146 | 0.167 ± 0.144 | 0.686 ± 0.449 | 0.244 ± 0.107 | 0.617 ± 0.281 | 0.390 ± 0.070 | 0.521 ± 0.168 |
| Switchgrass G3 HT | rrBLUP/ridge | 0.558 ± 0.071 | 0.839 ± 0.067 | 0.360 ± 0.164 | 1.333 ± 0.342 | 0.433 ± 0.149 | 1.052 ± 0.237 | 0.505 ± 0.097 | 0.854 ± 0.167 |

*Note. Values are mean ± SD across 25 outer-fold evaluation contexts (5 repeats × 5 folds). SD summarizes empirical fold-to-fold dispersion only; the contexts are correlated resampling evaluations and are not independent biological replicates.*

**Supplementary Table S2. Model choice reversal and conditional near-*r* discordance summary.**

| **Setting** | **k (%)** | **Contexts** | ***r*-best != recovery-best (%)** | ***r*-best != OSD-best (%)** | **Near-*r* pairs** | **Strict recovery discordance** | **Strict OSD discordance** |
| --- | --- | --- | --- | --- | --- | --- | --- |
| Cross-environment maize | 5 | 20 | 45 | 45 | 48 | 1/48 (2.1%) | 4/48 (8.3%) |
| Cross-environment maize | 10 | 20 | 55 | 45 | 48 | 0/48 (0.0%) | 1/48 (2.1%) |
| Cross-environment maize | 20 | 20 | 25 | 35 | 48 | 0/48 (0.0%) | 0/48 (0.0%) |
| Within-environment nested CV | 5 | 50 | 40 | 34 | 33 | 3/33 (9.1%) | 5/33 (15.2%) |
| Within-environment nested CV | 10 | 50 | 42 | 32 | 33 | 5/33 (15.2%) | 5/33 (15.2%) |
| Within-environment nested CV | 20 | 50 | 48 | 54 | 33 | 5/33 (15.2%) | 4/33 (12.1%) |

*Note. Strict discordance is conditioned on near-r comparisons. Raw numerators, denominators, and percentages are descriptive summaries of correlated evaluation contexts; no binomial confidence intervals are attached.*

**Supplementary Table S3. Predicted top-k overlap across model pairs, datasets, and selection intensities.**

| **Dataset** | **k (%)** | **Model pair** | **Contexts (n)** | **Mean \|Δ*r*\|** | **Mean Jaccard** | **Mean overlap fraction** | **SD overlap** | **Mean \|ΔRecovery\|** |
| --- | --- | --- | --- | --- | --- | --- | --- | --- |
| Maize NCII6210 EW | 5 | LightGBM vs MLP | 25 | 0.047 | 0.446 | 0.614 | 0.063 | 0.058 |
| Maize NCII6210 EW | 5 | LightGBM vs rrBLUP/ridge | 25 | 0.008 | 0.575 | 0.729 | 0.036 | 0.033 |
| Maize NCII6210 EW | 5 | MLP vs rrBLUP/ridge | 25 | 0.055 | 0.43 | 0.599 | 0.062 | 0.068 |
| Maize NCII6210 EW | 10 | LightGBM vs MLP | 25 | 0.047 | 0.508 | 0.672 | 0.052 | 0.05 |
| Maize NCII6210 EW | 10 | LightGBM vs rrBLUP/ridge | 25 | 0.008 | 0.621 | 0.766 | 0.03 | 0.023 |
| Maize NCII6210 EW | 10 | MLP vs rrBLUP/ridge | 25 | 0.055 | 0.484 | 0.651 | 0.042 | 0.048 |
| Maize NCII6210 EW | 20 | LightGBM vs MLP | 25 | 0.047 | 0.563 | 0.72 | 0.027 | 0.049 |
| Maize NCII6210 EW | 20 | LightGBM vs rrBLUP/ridge | 25 | 0.008 | 0.69 | 0.816 | 0.02 | 0.017 |
| Maize NCII6210 EW | 20 | MLP vs rrBLUP/ridge | 25 | 0.055 | 0.555 | 0.713 | 0.022 | 0.045 |
| Switchgrass G3 HT | 5 | LightGBM vs MLP | 25 | 0.269 | 0.19 | 0.293 | 0.211 | 0.2 |
| Switchgrass G3 HT | 5 | LightGBM vs rrBLUP/ridge | 25 | 0.034 | 0.603 | 0.74 | 0.128 | 0.073 |
| Switchgrass G3 HT | 5 | MLP vs rrBLUP/ridge | 25 | 0.246 | 0.192 | 0.3 | 0.198 | 0.207 |
| Switchgrass G3 HT | 10 | LightGBM vs MLP | 25 | 0.269 | 0.24 | 0.378 | 0.125 | 0.236 |
| Switchgrass G3 HT | 10 | LightGBM vs rrBLUP/ridge | 25 | 0.034 | 0.577 | 0.72 | 0.123 | 0.069 |
| Switchgrass G3 HT | 10 | MLP vs rrBLUP/ridge | 25 | 0.246 | 0.246 | 0.389 | 0.1 | 0.189 |
| Switchgrass G3 HT | 20 | LightGBM vs MLP | 25 | 0.269 | 0.279 | 0.432 | 0.084 | 0.11 |
| Switchgrass G3 HT | 20 | LightGBM vs rrBLUP/ridge | 25 | 0.034 | 0.565 | 0.718 | 0.071 | 0.069 |
| Switchgrass G3 HT | 20 | MLP vs rrBLUP/ridge | 25 | 0.246 | 0.308 | 0.467 | 0.08 | 0.13 |

*Note. Overlap fraction is the shared-candidate count divided by the selected set size. SD overlap describes empirical dispersion across correlated outer-fold evaluation contexts and is not inferential uncertainty.*

**Supplementary Table S4. Model implementation and hyperparameter settings.**

| **Evaluation setting** | **Model** | **Implementation** | **Tuning or fixed settings** |
| --- | --- | --- | --- |
| Within-environment nested CV | rrBLUP/ridge | scikit-learn Ridge in a StandardScaler pipeline | Inner 5-fold CV; alpha in {0.1, 1, 10, 100}; alpha = 100 selected in all final outer folds. |
| Within-environment nested CV | LightGBM | Gradient-boosted tree ensemble | Eight random candidates per outer fold from learning_rate {0.01, 0.03, 0.05, 0.1}; num_leaves {15, 31, 63}; min_child_samples {10, 20, 50}; subsample {0.70, 0.85, 1.00}; colsample_bytree {0.70, 0.85, 1.00}; n_estimators {100, 160, 240}. |
| Within-environment nested CV | MLP | scikit-learn multilayer perceptron | Four random candidates per outer fold from hidden_layer_sizes {(64,), (128,), (128,64), (256,128)}; learning_rate_init {1e-4, 5e-4, 1e-3}; alpha {1e-5, 1e-4, 1e-3}; batch_size {32, 64, 128}; ReLU; Adam; max_iter 80; early stopping; n_iter_no_change 10. |
| Cross-environment maize | rrBLUP/ridge | Ridge regression | Fixed alpha = 1.0. |
| Cross-environment maize | LightGBM | Gradient-boosted tree ensemble | Fixed learning_rate 0.05; max_depth 5; n_estimators 160; min_child_samples 20; num_leaves 10; subsample 1.0; colsample_bytree 1.0. |
| Cross-environment maize | MLP | Multilayer perceptron | Fixed hidden_layer_sizes (128,64); alpha 1e-4; learning_rate_init 1e-3; max_iter 80; early stopping; n_iter_no_change 10. |
| Cross-environment maize | MeNet | Mixed-effect deep neural network | For each directed pair, 20% of the training-environment samples were used for validation; no target-environment phenotypes were used in training. Batch size 16; 50 epochs; learning rate 0.001; dropout 0.3 in VE, RepGeno, and output components. |

*Note. Within-environment hyperparameters were selected exclusively within the outer training data by inner 5-fold cross-validation. Cross-environment rrBLUP/ridge, LightGBM, and MLP used fixed configurations across directed environment pairs; MLP early stopping and MeNet validation used source-training data only. No target-environment phenotypes were used for hyperparameter selection, model fitting, or validation.*

**Supplementary Table S5. Cross-environment metrics by selection intensity and model.**

| **k (%)** | **Model** | **Mean Pearson *r*** | **Mean observed top-k recovery** | **Mean Jaccard with observed top-k set** | **Mean OSD@k** |
| --- | --- | --- | --- | --- | --- |
| 5 | LightGBM | 0.408 | 0.202 | 0.114 | 0.754 |
| 5 | MLP | 0.345 | 0.173 | 0.095 | 0.701 |
| 5 | MeNet | 0.396 | 0.183 | 0.102 | 0.729 |
| 5 | rrBLUP | 0.402 | 0.193 | 0.108 | 0.748 |
| 10 | LightGBM | 0.408 | 0.270 | 0.158 | 0.641 |
| 10 | MLP | 0.345 | 0.250 | 0.144 | 0.581 |
| 10 | MeNet | 0.396 | 0.259 | 0.150 | 0.622 |
| 10 | rrBLUP | 0.402 | 0.270 | 0.158 | 0.648 |
| 20 | LightGBM | 0.408 | 0.381 | 0.237 | 0.523 |
| 20 | MLP | 0.345 | 0.358 | 0.219 | 0.466 |
| 20 | MeNet | 0.396 | 0.376 | 0.233 | 0.514 |
| 20 | rrBLUP | 0.402 | 0.376 | 0.233 | 0.520 |

*Note. Values are means across the 20 directed maize train-test environment pairs. The source outputs used the labels Recall@k and Gain@k; in the manuscript these are described as observed top-k recovery and OSD@k, respectively.*

**Supplementary Table S6. Marker quality-control summary for the processed input matrices.**

| **Dataset** | **Samples (n)** | **Markers (n)** | **Total missing rate** | **Maximum marker missing rate** | **Minimum MAF** | **Median MAF** |
| --- | --- | --- | --- | --- | --- | --- |
| Maize NCII6210 EW | 5831 | 5000 | 0.000 | 0.000 | 0.0539 | 0.2296 |
| Switchgrass G3 HT | 514 | 10000 | 0.000 | 0.000 | 0.0545 | 0.4553 |

*Note. Statistics refer to the final processed genotype matrices used in the within-environment analyses. MAF = minor allele frequency.*

**Supplementary Table S7. Near-*r* discordance mechanism examples.**

| **Setting** | **Dataset** | **Context** | **k (%)** | **Model pair** | **\|Δ*r*\|** | **\|ΔRecovery\|** | **\|ΔOSD\|** | **Top-k overlap** | **Jaccard** | **Whole-sample rank ρ** | **Upper-tail rank ρ** | **Mean abs tail rank shift (percentile points)** | **Selected n/model** | **Non-shared n/model** | **Interpretation** |
| --- | --- | --- | --- | --- | --- | --- | --- | --- | --- | --- | --- | --- | --- | --- | --- |
| within_environment | switchgrass_G3_HT | repeat1_fold4 | 5 | LightGBM vs rrBLUP/ridge | 0.000626471 | 0 | 0.628286 | 0.666667 | 0.5 |  |  |  |  |  | Same recovery, large OSD difference |
| within_environment | switchgrass_G3_HT | repeat3_fold4 | 5 | LightGBM vs rrBLUP/ridge | 0.0111829 | 0.166667 | 0.378822 | 0.5 | 0.333333 |  |  |  |  |  | Different phenotype-defined top-k recovery and candidate identities |
| within_environment | switchgrass_G3_HT | repeat5_fold3 | 5 | LightGBM vs rrBLUP/ridge | 0.000504567 | 0 | 0.0803417 | 0.5 | 0.333333 |  |  |  |  |  | Near-identical *r* but only half of top-5% candidates shared |
| within_environment | switchgrass_G3_HT | repeat5_fold3 | 10 | LightGBM vs rrBLUP/ridge | 0.000504567 | 0.181818 | 0.356995 | 0.545455 | 0.375 |  |  |  |  |  | Near-identical *r* with recovery and composition differences |
| cross_environment | Maize_5loc_EW | HeB_to_BJ | 5 | LightGBM vs rrBLUP | 0.0109392 | 0.0652921 | 0.155916 | 0.549828 | 0.379147 | 0.871319 | 0.0983456 | 6.6967 | 291 | 131 | Global predictions remain strongly rank-correlated, but the top tail is weakly concordant; candidate turnover drives OSD discordance. |
| cross_environment | Maize_5loc_EW | BJ_to_LN | 5 | LightGBM vs MLP | 0.00987545 | 0.0344828 | 0.252829 | 0.472414 | 0.309255 | 0.801106 | -0.0715166 | 8.76862 | 290 | 153 | Near-*r* models show substantial top-tail reordering despite moderate-to-high whole-sample rank similarity. |

*Note. These case-level diagnostics distinguish candidate set turnover and upper-tail rank rearrangement from wholesale disagreement across the full prediction distribution. Blank rank-correlation fields indicate that exact synchronized prediction-level outputs were not used for that within-environment case.*

**Supplementary Table S8. Sensitivity of strict discordance rates to operational thresholds.**

| **Panel** | **Setting** | **k (%)** | **Near-*r* cutoff \|Δ*r*\|** | **Recovery cutoff \|ΔRecovery\|** | **OSD cutoff \|ΔOSD\|** | **Near-*r* pairs** | **Strict recovery** | **Strict OSD** |
| --- | --- | --- | --- | --- | --- | --- | --- | --- |
| A. Near-*r* threshold sensitivity | Within-environment nested CV | 5 | 0.01 | 0.08 | 0.15 | 22 | 1/22 (4.5%) | 3/22 (13.6%) |
| A. Near-*r* threshold sensitivity | Within-environment nested CV | 5 | 0.02 | 0.08 | 0.15 | 33 | 3/33 (9.1%) | 5/33 (15.2%) |
| A. Near-*r* threshold sensitivity | Within-environment nested CV | 5 | 0.05 | 0.08 | 0.15 | 73 | 16/73 (21.9%) | 17/73 (23.3%) |
| B. Recovery discordance cutoff sensitivity | Within-environment nested CV | 5 | 0.02 | 0.05 |  | 33 | 12/33 (36.4%) |  |
| B. Recovery discordance cutoff sensitivity | Within-environment nested CV | 5 | 0.02 | 0.08 |  | 33 | 3/33 (9.1%) |  |
| B. Recovery discordance cutoff sensitivity | Within-environment nested CV | 5 | 0.02 | 0.1 |  | 33 | 3/33 (9.1%) |  |
| C. OSD discordance cutoff sensitivity | Within-environment nested CV | 5 | 0.02 |  | 0.1 | 33 |  | 8/33 (24.2%) |
| C. OSD discordance cutoff sensitivity | Within-environment nested CV | 5 | 0.02 |  | 0.15 | 33 |  | 5/33 (15.2%) |
| C. OSD discordance cutoff sensitivity | Within-environment nested CV | 5 | 0.02 |  | 0.2 | 33 |  | 5/33 (15.2%) |
| A. Near-*r* threshold sensitivity | Within-environment nested CV | 10 | 0.01 | 0.08 | 0.15 | 22 | 2/22 (9.1%) | 2/22 (9.1%) |
| A. Near-*r* threshold sensitivity | Within-environment nested CV | 10 | 0.02 | 0.08 | 0.15 | 33 | 5/33 (15.2%) | 5/33 (15.2%) |
| A. Near-*r* threshold sensitivity | Within-environment nested CV | 10 | 0.05 | 0.08 | 0.15 | 73 | 15/73 (20.5%) | 9/73 (12.3%) |
| B. Recovery discordance cutoff sensitivity | Within-environment nested CV | 10 | 0.02 | 0.05 |  | 33 | 8/33 (24.2%) |  |
| B. Recovery discordance cutoff sensitivity | Within-environment nested CV | 10 | 0.02 | 0.08 |  | 33 | 5/33 (15.2%) |  |
| B. Recovery discordance cutoff sensitivity | Within-environment nested CV | 10 | 0.02 | 0.1 |  | 33 | 1/33 (3.0%) |  |
| C. OSD discordance cutoff sensitivity | Within-environment nested CV | 10 | 0.02 |  | 0.1 | 33 |  | 7/33 (21.2%) |
| C. OSD discordance cutoff sensitivity | Within-environment nested CV | 10 | 0.02 |  | 0.15 | 33 |  | 5/33 (15.2%) |
| C. OSD discordance cutoff sensitivity | Within-environment nested CV | 10 | 0.02 |  | 0.2 | 33 |  | 3/33 (9.1%) |
| A. Near-*r* threshold sensitivity | Within-environment nested CV | 20 | 0.01 | 0.08 | 0.15 | 22 | 3/22 (13.6%) | 2/22 (9.1%) |
| A. Near-*r* threshold sensitivity | Within-environment nested CV | 20 | 0.02 | 0.08 | 0.15 | 33 | 5/33 (15.2%) | 4/33 (12.1%) |
| A. Near-*r* threshold sensitivity | Within-environment nested CV | 20 | 0.05 | 0.08 | 0.15 | 73 | 15/73 (20.5%) | 8/73 (11.0%) |
| B. Recovery discordance cutoff sensitivity | Within-environment nested CV | 20 | 0.02 | 0.05 |  | 33 | 5/33 (15.2%) |  |
| B. Recovery discordance cutoff sensitivity | Within-environment nested CV | 20 | 0.02 | 0.08 |  | 33 | 5/33 (15.2%) |  |
| B. Recovery discordance cutoff sensitivity | Within-environment nested CV | 20 | 0.02 | 0.1 |  | 33 | 2/33 (6.1%) |  |
| C. OSD discordance cutoff sensitivity | Within-environment nested CV | 20 | 0.02 |  | 0.1 | 33 |  | 6/33 (18.2%) |
| C. OSD discordance cutoff sensitivity | Within-environment nested CV | 20 | 0.02 |  | 0.15 | 33 |  | 4/33 (12.1%) |
| C. OSD discordance cutoff sensitivity | Within-environment nested CV | 20 | 0.02 |  | 0.2 | 33 |  | 4/33 (12.1%) |
| A. Near-*r* threshold sensitivity | Cross-environment maize | 5 | 0.01 | 0.08 | 0.15 | 25 | 0/25 (0.0%) | 2/25 (8.0%) |
| A. Near-*r* threshold sensitivity | Cross-environment maize | 5 | 0.02 | 0.08 | 0.15 | 48 | 1/48 (2.1%) | 4/48 (8.3%) |
| A. Near-*r* threshold sensitivity | Cross-environment maize | 5 | 0.05 | 0.08 | 0.15 | 92 | 3/92 (3.3%) | 7/92 (7.6%) |
| B. Recovery discordance cutoff sensitivity | Cross-environment maize | 5 | 0.02 | 0.05 |  | 48 | 3/48 (6.2%) |  |
| B. Recovery discordance cutoff sensitivity | Cross-environment maize | 5 | 0.02 | 0.08 |  | 48 | 1/48 (2.1%) |  |
| B. Recovery discordance cutoff sensitivity | Cross-environment maize | 5 | 0.02 | 0.1 |  | 48 | 0/48 (0.0%) |  |
| C. OSD discordance cutoff sensitivity | Cross-environment maize | 5 | 0.02 |  | 0.1 | 48 |  | 10/48 (20.8%) |
| C. OSD discordance cutoff sensitivity | Cross-environment maize | 5 | 0.02 |  | 0.15 | 48 |  | 4/48 (8.3%) |
| C. OSD discordance cutoff sensitivity | Cross-environment maize | 5 | 0.02 |  | 0.2 | 48 |  | 2/48 (4.2%) |
| A. Near-*r* threshold sensitivity | Cross-environment maize | 10 | 0.01 | 0.08 | 0.15 | 25 | 0/25 (0.0%) | 1/25 (4.0%) |
| A. Near-*r* threshold sensitivity | Cross-environment maize | 10 | 0.02 | 0.08 | 0.15 | 48 | 0/48 (0.0%) | 1/48 (2.1%) |
| A. Near-*r* threshold sensitivity | Cross-environment maize | 10 | 0.05 | 0.08 | 0.15 | 92 | 0/92 (0.0%) | 3/92 (3.3%) |
| B. Recovery discordance cutoff sensitivity | Cross-environment maize | 10 | 0.02 | 0.05 |  | 48 | 1/48 (2.1%) |  |
| B. Recovery discordance cutoff sensitivity | Cross-environment maize | 10 | 0.02 | 0.08 |  | 48 | 0/48 (0.0%) |  |
| B. Recovery discordance cutoff sensitivity | Cross-environment maize | 10 | 0.02 | 0.1 |  | 48 | 0/48 (0.0%) |  |
| C. OSD discordance cutoff sensitivity | Cross-environment maize | 10 | 0.02 |  | 0.1 | 48 |  | 5/48 (10.4%) |
| C. OSD discordance cutoff sensitivity | Cross-environment maize | 10 | 0.02 |  | 0.15 | 48 |  | 1/48 (2.1%) |
| C. OSD discordance cutoff sensitivity | Cross-environment maize | 10 | 0.02 |  | 0.2 | 48 |  | 0/48 (0.0%) |
| A. Near-*r* threshold sensitivity | Cross-environment maize | 20 | 0.01 | 0.08 | 0.15 | 25 | 0/25 (0.0%) | 0/25 (0.0%) |
| A. Near-*r* threshold sensitivity | Cross-environment maize | 20 | 0.02 | 0.08 | 0.15 | 48 | 0/48 (0.0%) | 0/48 (0.0%) |
| A. Near-*r* threshold sensitivity | Cross-environment maize | 20 | 0.05 | 0.08 | 0.15 | 92 | 0/92 (0.0%) | 0/92 (0.0%) |
| B. Recovery discordance cutoff sensitivity | Cross-environment maize | 20 | 0.02 | 0.05 |  | 48 | 0/48 (0.0%) |  |
| B. Recovery discordance cutoff sensitivity | Cross-environment maize | 20 | 0.02 | 0.08 |  | 48 | 0/48 (0.0%) |  |
| B. Recovery discordance cutoff sensitivity | Cross-environment maize | 20 | 0.02 | 0.1 |  | 48 | 0/48 (0.0%) |  |
| C. OSD discordance cutoff sensitivity | Cross-environment maize | 20 | 0.02 |  | 0.1 | 48 |  | 0/48 (0.0%) |
| C. OSD discordance cutoff sensitivity | Cross-environment maize | 20 | 0.02 |  | 0.15 | 48 |  | 0/48 (0.0%) |
| C. OSD discordance cutoff sensitivity | Cross-environment maize | 20 | 0.02 |  | 0.2 | 48 |  | 0/48 (0.0%) |

*Note. Near-r and strict discordance cutoffs are operational filters. Conditional percentages vary in magnitude as thresholds change and should not be interpreted as universal biological decision boundaries.*

**Supplementary Table S9. Selection confusion-matrix metrics under fixed-cardinality top-k selection.**

| **Setting** | **Dataset** | **Model** | **k (%)** | **Evaluations** | **Mean selected n** | **Mean TP** | **Mean FP = FN** | **Mean TN** | **Recovery = Precision = Recall = F1** | **Specificity** |
| --- | --- | --- | --- | --- | --- | --- | --- | --- | --- | --- |
| Within-environment nested CV | Maize NCII6210 EW | rrBLUP/ridge | 5 | 25 | 59 | 28.2 | 30.8 | 1076.4 | 0.479 ± 0.049 | 0.972 ± 0.003 |
| Within-environment nested CV | Maize NCII6210 EW | LightGBM | 5 | 25 | 59 | 27.6 | 31.4 | 1075.8 | 0.468 ± 0.046 | 0.972 ± 0.002 |
| Within-environment nested CV | Maize NCII6210 EW | MLP | 5 | 25 | 59 | 24.5 | 34.5 | 1072.7 | 0.416 ± 0.063 | 0.969 ± 0.003 |
| Within-environment nested CV | Maize NCII6210 EW | rrBLUP/ridge | 10 | 25 | 117 | 62.5 | 54.5 | 994.7 | 0.534 ± 0.033 | 0.948 ± 0.004 |
| Within-environment nested CV | Maize NCII6210 EW | LightGBM | 10 | 25 | 117 | 62.6 | 54.4 | 994.8 | 0.535 ± 0.027 | 0.948 ± 0.003 |
| Within-environment nested CV | Maize NCII6210 EW | MLP | 10 | 25 | 117 | 57.3 | 59.7 | 989.5 | 0.490 ± 0.036 | 0.943 ± 0.004 |
| Within-environment nested CV | Maize NCII6210 EW | rrBLUP/ridge | 20 | 25 | 234 | 142.6 | 91.4 | 840.8 | 0.610 ± 0.025 | 0.902 ± 0.006 |
| Within-environment nested CV | Maize NCII6210 EW | LightGBM | 20 | 25 | 234 | 142.9 | 91.1 | 841.1 | 0.611 ± 0.028 | 0.902 ± 0.007 |
| Within-environment nested CV | Maize NCII6210 EW | MLP | 20 | 25 | 234 | 132.1 | 101.9 | 830.3 | 0.565 ± 0.025 | 0.891 ± 0.006 |
| Within-environment nested CV | Switchgrass G3 HT | rrBLUP/ridge | 5 | 25 | 6 | 2.2 | 3.8 | 93 | 0.360 ± 0.164 | 0.960 ± 0.010 |
| Within-environment nested CV | Switchgrass G3 HT | LightGBM | 5 | 25 | 6 | 2.1 | 3.9 | 92.9 | 0.353 ± 0.200 | 0.960 ± 0.012 |
| Within-environment nested CV | Switchgrass G3 HT | MLP | 5 | 25 | 6 | 1 | 5 | 91.8 | 0.167 ± 0.144 | 0.948 ± 0.009 |
| Within-environment nested CV | Switchgrass G3 HT | rrBLUP/ridge | 10 | 25 | 11 | 4.8 | 6.2 | 85.6 | 0.433 ± 0.149 | 0.932 ± 0.018 |
| Within-environment nested CV | Switchgrass G3 HT | LightGBM | 10 | 25 | 11 | 5.1 | 5.9 | 85.9 | 0.465 ± 0.182 | 0.936 ± 0.022 |
| Within-environment nested CV | Switchgrass G3 HT | MLP | 10 | 25 | 11 | 2.7 | 8.3 | 83.5 | 0.244 ± 0.107 | 0.909 ± 0.013 |
| Within-environment nested CV | Switchgrass G3 HT | rrBLUP/ridge | 20 | 25 | 21 | 10.6 | 10.4 | 71.4 | 0.505 ± 0.097 | 0.873 ± 0.025 |
| Within-environment nested CV | Switchgrass G3 HT | LightGBM | 20 | 25 | 21 | 10.4 | 10.6 | 71.2 | 0.493 ± 0.070 | 0.870 ± 0.018 |
| Within-environment nested CV | Switchgrass G3 HT | MLP | 20 | 25 | 21 | 8.2 | 12.8 | 69 | 0.390 ± 0.070 | 0.844 ± 0.018 |
| Cross-environment directed | Maize NCII6210 5-location EW | rrBLUP | 5 | 20 | 290 | 56 | 234 | 5266.6 | 0.193 ± 0.056 | 0.957 ± 0.003 |
| Cross-environment directed | Maize NCII6210 5-location EW | LightGBM | 5 | 20 | 290 | 58.5 | 231.5 | 5269.1 | 0.202 ± 0.066 | 0.958 ± 0.003 |
| Cross-environment directed | Maize NCII6210 5-location EW | MeNet | 5 | 20 | 290 | 53.2 | 236.8 | 5263.8 | 0.183 ± 0.058 | 0.957 ± 0.003 |
| Cross-environment directed | Maize NCII6210 5-location EW | MLP | 5 | 20 | 290 | 50.2 | 239.8 | 5260.8 | 0.173 ± 0.045 | 0.956 ± 0.002 |
| Cross-environment directed | Maize NCII6210 5-location EW | rrBLUP | 10 | 20 | 579.4 | 156.6 | 422.8 | 4788.4 | 0.270 ± 0.062 | 0.919 ± 0.007 |
| Cross-environment directed | Maize NCII6210 5-location EW | LightGBM | 10 | 20 | 579.4 | 156.2 | 423.2 | 4788 | 0.270 ± 0.069 | 0.919 ± 0.008 |
| Cross-environment directed | Maize NCII6210 5-location EW | MeNet | 10 | 20 | 579.4 | 150.2 | 429.2 | 4782 | 0.259 ± 0.062 | 0.918 ± 0.007 |
| Cross-environment directed | Maize NCII6210 5-location EW | MLP | 10 | 20 | 579.4 | 145.1 | 434.3 | 4776.9 | 0.250 ± 0.053 | 0.917 ± 0.006 |
| Cross-environment directed | Maize NCII6210 5-location EW | rrBLUP | 20 | 20 | 1158.4 | 435.8 | 722.6 | 3909.6 | 0.376 ± 0.061 | 0.844 ± 0.015 |
| Cross-environment directed | Maize NCII6210 5-location EW | LightGBM | 20 | 20 | 1158.4 | 441.4 | 717 | 3915.2 | 0.381 ± 0.067 | 0.845 ± 0.017 |
| Cross-environment directed | Maize NCII6210 5-location EW | MeNet | 20 | 20 | 1158.4 | 435 | 723.4 | 3908.8 | 0.376 ± 0.063 | 0.844 ± 0.016 |
| Cross-environment directed | Maize NCII6210 5-location EW | MLP | 20 | 20 | 1158.4 | 414.8 | 743.6 | 3888.6 | 0.358 ± 0.055 | 0.840 ± 0.014 |

*Note. Because the observed and predicted positive sets have the same size, FP = FN and Recovery@k = Precision = Recall = F1 by construction. Values shown as mean ± SD are descriptive across correlated evaluation contexts.*

**Supplementary Table S10. Prediction equations, intercept/bias terms, and scaling by model and evaluation setting.**

| **Setting** | **Model** | **Prediction equation (conceptual)** | **Genotype scaling** | **Response used for fitting** | **Intercept / bias** | **Scale used for evaluation** |
| --- | --- | --- | --- | --- | --- | --- |
| Within-environment nested CV | rrBLUP/ridge | ẑ = b₀ + Σⱼ βⱼ x̃ⱼ; ŷ = training mean + training SD × ẑ | Training-fold StandardScaler (mean 0, SD 1 per marker); applied to validation/test | z = (y − training mean) / training SD | Fitted Ridge intercept retained; no post-hoc test-set intercept adjustment | Back-transformed to original processed phenotype scale before all metrics |
| Within-environment nested CV | LightGBM | ẑ = F(x̃), where F is the boosted-tree ensemble; ŷ = training mean + training SD × ẑ | Training-fold StandardScaler in the final nested-CV script | z = (y − training mean) / training SD | No separate linear intercept specified; boosting baseline/tree ensemble handled internally | Back-transformed to original processed phenotype scale before all metrics |
| Within-environment nested CV | MLP | Feedforward ReLU hidden layers with learned biases; scalar standardized output ẑ; ŷ = training mean + training SD × ẑ | Training-fold StandardScaler; applied to validation/test | z = (y − training mean) / training SD | Learned neural bias terms; no post-hoc test-set intercept adjustment | Back-transformed to original processed phenotype scale before all metrics |
| Directed cross-environment | rrBLUP/ridge | Source-scale ẑ = b₀ + Σⱼ βⱼ x̃ⱼ | StandardScaler fitted on source-environment genotypes; applied to target genotypes | Source-environment z score: (y − source mean) / source SD | Fitted Ridge intercept retained; no target-label recalibration | Prediction retained on source-standardized response scale; target observed EW independently standardized within target environment |
| Directed cross-environment | LightGBM | Source-scale ẑ = F(x), where F is the boosted-tree ensemble | Raw numeric marker inputs in the archived cross-environment script (no StandardScaler) | Source-environment z score: (y − source mean) / source SD | No separate linear intercept specified; boosting baseline/tree ensemble handled internally; no target-label recalibration | Prediction retained on source-standardized response scale; target observed EW independently standardized within target environment |
| Directed cross-environment | MLP | Feedforward ReLU hidden layers with learned biases; source-scale scalar output ẑ | StandardScaler fitted on source-environment genotypes; applied to target genotypes | Source-environment z score: (y − source mean) / source SD | Learned neural bias terms; no target-label recalibration | Prediction retained on source-standardized response scale; target observed EW independently standardized within target environment |
| Directed cross-environment | MeNet | ẑ = fθ(genotype, RepGeno / genetic-relatedness representation), ending in a scalar neural output | Genotype states recoded 0/1/2 to -1/0/1 for the final MeNet preparation | Environment-standardized phenotype files in the final server-side run | Learned bias terms in neural linear layers; no post-hoc target-label intercept calibration | Standardized scale prediction; target y_true in archived pairwise outputs has mean ~0 and population SD ~1 |

*Note. Within-environment predictions were back-transformed to the original processed phenotype scale before metrics were calculated. Cross-environment predictions were not post-hoc recalibrated using target-environment phenotypes; cross-environment RMSE is therefore a standardized scale error.*

Supplementary Table S11. Representative near-*r* strict discordance cases and decision-level decomposition.

| **Case** | **Setting / context** | **k (%)** | **Model pair** | ***r* (A / B)** | **\|Δ*r*\|** | **Recovery (A / B)** | **\|ΔRecovery\|** | **OSD (A / B)** | **\|ΔOSD\|** | **Top-k overlap** | **Whole-sample rank ρ** | **Upper-tail rank ρ** | **Non-shared n/model** |
| --- | --- | --- | --- | --- | --- | --- | --- | --- | --- | --- | --- | --- | --- |
| 1. composition discordance with identical recovery | Switchgrass; repeat 1, fold 4 | 5 | LightGBM vs rrBLUP/ridge | 0.458 / 0.458 | 0.0006 | 0.333 / 0.333 | 0 | 1.626 / 0.997 | 0.628 | 0.667 |  |  | 2 |
| 2. recovery and composition discordance | Switchgrass; repeat 3, fold 4 | 5 | LightGBM vs rrBLUP/ridge | 0.668 / 0.656 | 0.0112 | 0.333 / 0.500 | 0.167 | 1.422 / 1.801 | 0.379 | 0.5 |  |  | 3 |
| 3. cross-environment upper-tail rearrangement | Maize HeB→BJ; HeB->BJ | 5 | LightGBM vs rrBLUP | 0.632 / 0.621 | 0.0109 | 0.344 / 0.278 | 0.065 | 1.243 / 1.088 | 0.156 | 0.55 | 0.871 | 0.098 | 131 |

*Note. Representative cases were selected to illustrate composition discordance, recovery-plus-composition discordance, and cross-environment upper-tail rank rearrangement. These analyses demonstrate decision-level mechanisms but do not identify causal SNPs or genomic regions.*

**Supplementary Notes**

**Tie handling**

The archived implementation retains exactly the fixed candidate set cardinality defined in Methods Section 2.6 after descending sorting and does not expand the selected set when values are tied at the cutoff. In the archived prediction-level audit, no model prediction ties were detected at the evaluated top-k boundaries. Phenotype cutoff ties were uncommon and involved only small tied groups. The fixed-cardinality rule is retained for reproducibility.

**Interpretation of phenotype-referenced diagnostics**

Observed top-k recovery and OSD@k are evaluated against observed or adjusted phenotypes, not known true breeding values. They should therefore be interpreted as retrospective phenotype-referenced candidate diagnostics rather than estimates of realized genetic gain or response to selection in a subsequent generation.

**Dependence of evaluation contexts**

Repeated outer-fold contexts reuse individuals across repeats, and directed environment pairs share environments and germplasm. Fold- and pair-level summaries are therefore treated descriptively rather than as independent biological replicates. SD values describe empirical dispersion; no population-level inference is based on nominal fold or directed-pair counts.
